# The future burden of type 2 diabetes in Belgium: a microsimulation model

**DOI:** 10.1186/s12963-024-00328-y

**Published:** 2024-04-23

**Authors:** Elly Mertens, Junior Ocira, Diana Sagastume, Maria Salve Vasquez, Stefanie Vandevijvere, José L. Peñalvo

**Affiliations:** 1 Unit of Non-Communicable Diseases, Department of Public Health, Institute of Tropical Medicine, Antwerp, Belgium; 2 Access-To-Medicines Research Centre, Faculty of Economics and Business, KU Leuven, Louvain, Belgium; 3 Department of Epidemiology and Public Health, Service of Health Information, Sciensano, Brussels, Belgium; 4 Global Health Institute, University of Antwerp, Antwerp, Belgium; 5 National Center for Epidemiology, Instituto de Salud Carlos III, Madrid, Spain

**Keywords:** Diabetes, Microsimulation, Burden, Forecast

## Abstract

**Objective:**

To forecast the annual burden of type 2 diabetes and related socio-demographic disparities in Belgium until 2030.

**Methods:**

This study utilized a discrete-event transition microsimulation model. A synthetic population was created using 2018 national register data of the Belgian population aged 0–80 years, along with the national representative prevalence of diabetes risk factors obtained from the latest (2018) Belgian Health Interview and Examination Surveys using Multiple Imputation by Chained Equations (MICE) as inputs to the Simulation of Synthetic Complex Data (simPop) model. Mortality information was obtained from the Belgian vital statistics and used to calculate annual death probabilities. From 2018 to 2030, synthetic individuals transitioned annually from health to death, with or without developing type 2 diabetes, as predicted by the Finnish Diabetes Risk Score, and risk factors were updated via strata-specific transition probabilities.

**Results:**

A total of 6722 [95% UI 3421, 11,583] new cases of type 2 diabetes per 100,000 inhabitants are expected between 2018 and 2030 in Belgium, representing a 32.8% and 19.3% increase in T2D prevalence rate and DALYs rate, respectively. While T2D burden remained highest for lower-education subgroups across all three Belgian regions, the highest increases in incidence and prevalence rates by 2030 are observed for women in general, and particularly among Flemish women reporting higher-education levels with a 114.5% and 44.6% increase in prevalence and DALYs rates, respectively. Existing age- and education-related inequalities will remain apparent in 2030 across all three regions.

**Conclusions:**

The projected increase in the burden of T2D in Belgium highlights the urgent need for primary and secondary preventive strategies. While emphasis should be placed on the lower-education groups, it is also crucial to reinforce strategies for people of higher socioeconomic status as the burden of T2D is expected to increase significantly in this population segment.

**Supplementary Information:**

The online version contains supplementary material available at 10.1186/s12963-024-00328-y.

## Introduction

Diabetes is a significant public health challenge in Europe, affecting around 61 million people (1 in 11 adults aged 20–79 years) in the European region in 2021 with nearly 90% of cases attributed to type 2 diabetes (T2D) [1]. In this region, there is a noticeable North–South gradient in diabetes prevalence, with age-adjusted prevalence rates among adults aged 20–79 years old ranging from 3% (32% undiagnosed) in Ireland to 15% (41%) in Turkey, as of 2021 [2]. T2D is a chronic metabolic disorder that is characterized by chronically elevated blood glucose resulting from a combination of insufficient insulin secretion and/or insulin resistance. Largely undiagnosed [2], uncontrolled hyperglycaemia and subsequent T2D often lead to a range of microvascular (e.g. neuro- and retinopathy) and macrovascular (e.g. cardiovascular diseases) complications that contribute significantly to morbidity and early mortality. According to the Global Burden of Disease (GBD) Study, T2D-related morbidity, measured as years lived with disability (YLD), is one of the highest-ranked health burdens in Europe. In 2019, T2D was estimated to have caused 4.4 million YLD in the European region, representing a 24% increase from 1990 [1]. This rise in T2D-related disability certainly reflects factors such as population aging [3], but also the increasing prevalence of risk factors for T2D, including excess weight, physical inactivity, unhealthy diet, and smoking [4], and their complex interactions.

Given the significant public health implications of the increasing burden of T2D, it is essential to closely monitor and accurately forecast its magnitude, particularly among potentially vulnerable socio-demographic strata. It is key to provide evidence-based information for public health preparedness, and design targeted and effective preventive strategies to reduce the impact of T2D on population health. Policy strategies for preventing and managing chronic non-communicable diseases (NCDs), including T2D, have increasingly relied on health decision modelling tools [5]. These tools, such as comparative risk assessments [6] or state-transition models [7], enable the evaluation of population-level health and economic impacts associated with potential public health intervention strategies. In particular, state-transition models, including both cohort models (e.g. Markov) and individual-based models (e.g. microsimulation), simulate consecutive trajectories across pre-defined health states [8]. This enables a prospective assessment of the disease burden and public health strategies. These models are valuable for informing policy decisions by providing insights into the long-term effects of continuing current trends versus trends resulting from potential preventive interventions. Individual-based microsimulation models have the added advantage of accounting for baseline variability in individual characteristics, risk profiles, and disease histories, which can influence the likelihood of health states occurring at specific time intervals [7, 9, 10]. By proactively evaluating future health outcomes for each individual, microsimulation models are considered a valuable tool for identifying drivers of health inequalities [11] and developing (cost-)effective population health strategies. Overall, individual-based microsimulation models provide a comprehensive approach to predicting disease outcomes, informing policy decisions, and promoting health equity [7].

The main aim of this study was to develop and validate a microsimulation model (T2D-M) for forecasting the future burden of T2D in Belgium while accounting for the population’s heterogeneity in individual, socioeconomic, and geographical layers. Further, the future burden was estimated across relevant socio-demographic strata (e.g., age, sex, region, and education level), allowing the identification of disparities and vulnerable groups within the Belgian population for supporting equitable health promotion programmes.

## Methods

### Model overview

The future burden of T2D was estimated using a life-course discrete-event state-transition microsimulation model describing the onset or progression of T2D individually and over the modelling time, incorporating socio-demographic factors and metabolic risk factors. Utilizing national representative distributions of socio-economic factors and T2D risk factors, including body mass index (BMI), waist circumference, blood pressure medication usage, and high blood glucose levels, the model recreates the Belgian population as a synthetic population. Each synthetic individual undergoes probabilistic transitions through the life-course, updating attributes and risk factors as they age, and predicting events until death or the end of the simulation period. These transitions occur annually, incorporating updates to risk factors, T2D status, and survival status through specific stochastic rules. The model's output provides estimates of T2D incidence, prevalence, and disability-adjusted life years (DALYs) in the synthetic population. Elements of the model population, model simulation, outputs, and uncertainty, including input sources for development and validation, are briefly described below and in detail in the Additional file 1: Technical Annex.

### Synthetic population

The T2D-M is based on a synthetic cohort that recreates the demographic characteristics and risk factors for T2D of the Belgian population. This cohort is informed by data obtained from the National Register and representative surveys, encompassing a wide range of socio-demographic factors. Belgium is a federal state that consists of three regions: the Brussels-Capital region, Flanders in the north, and Wallonia in the south. Additionally, Flanders and Wallonia are further subdivided into five provinces each. These distinctions have been introduced in the development of our synthetic cohort. As of January 1, 2018, the legal population of Belgium was 11,376,069 inhabitants [12]. The synthetic population was constructed using a set of variables including demographics (age, sex, province, region, education and income level), and risk factors (body mass index (BMI), waist circumference, use of BP medication, history of high blood glucose, and T2D status at present). This set of variables was obtained from a representative (age, sex, province) sample of the Belgian population provided by the latest (2018) Belgian Health Interview Survey (BHIS) [13] complemented with the latest (2018) Belgian Health Examination Survey (BELHES) [14] after multiple imputation by chained equations, implemented with the R package *mice* [15]. The fully informed survey data were further matched to the aggregated total of the Belgian population structure reported in the National Register of 2018 [12] using the R package Simulation of Synthetic Complex Data *SimPop* [16]. This implementation performs weighted sampling with replacement of individuals in each age group from the fully informed survey until reaching the total in the population for a specific age, and therefore ensuring that the correlation structure between variables and risk factors distributions were embedded in the starting model population.

### Model simulation

The T2D-M operates as a discrete-event state transition model with annual updates of the risk factors, T2D status and survival status of the individuals in the synthetic population. Starting from 2018 and annually, each synthetic individual enters the model according to baseline characteristics and then transitions across events from health to death, with or without developing T2D, simulated annually according to updated risk factors until death or the end of the simulation period in 2030.

### Update of risk factors

Advancing the simulation progress in annual cycles, the age of the synthetic individual is increased by one year in each cycle, while all other socio-demographic characteristics (such as sex, province of residence, and education level) remained unchanged. Subsequently, the modifiable risk factors for predicting T2D outlined in the concise model of the Finnish Diabetes Risk Score (FINDRISC) including BMI, waist circumference, use of blood pressure medication and history of high blood glucose [17] were updated based on the net annual transition probabilities, which were calculated using the simplex optimization algorithm, and age-smoothened prevalence of the risk factor states [18]. In each simulation year, and for every modifiable risk factor, the decision to transition to another risk factor state (e.g. from normal BMI to overweight) is determined by drawing from a bi- or multinomial distribution that incorporates strata-specific net-transition probabilities.

### Transitions between health states

The T2D-M incorporates three general health states: 1) health (free from T2D), 2) T2D, and 3) death, with the following potential transitions: from health to T2D, from T2D to death (caused by either T2D or other factors), and from health directly to death (caused only by other factors). At any given point in time, the synthetic individual can be in either the health, T2D, or death state. Once an individual transition to the T2D state, there is no possibility of returning to the health state. Furthermore, an individual can experience both T2D and death in the same year. Whether or not an individual transition to a new health state (i.e. T2D and/or death) in a particular year is dependent on a random draw from a binomial distribution. The probabilities for these transitions are determined based on the individual's risk profile. For each individual in the health state, their annual probability of developing T2D was calculated using the FINDRISC equation (17). This calculation considers the individual's prevalent risk factors, which are updated using the strata-specific transition probabilities outlined earlier. As the FINDRISC equation was originally designed to estimate an individual's 10-year risk of developing T2D, it was necessary to transform this value into a one-year probability [19] in order to appropriately use it in the simulation. Additionally, each individual's probability of death was calculated annually using strata-specific death probabilities. These probabilities were determined based on mortality rates obtained from the Standardised Procedures for Mortality Analysis (SPMA) database, the Belgian vital statistics collected annually from 1987 onwards [20]. For healthy individuals, the probability of death only considered the risk of death from causes other than T2D. However, for individuals with T2D, who are known to have an increased risk of all-cause mortality, probabilities of death from all causes were adjusted using age- and sex-specific relative risks derived from the Diabetes Epidemiology: Collaborative analysis of Diagnostic criteria in Europe (DECODE) study [21], a large population-based cohort in European populations, to obtain a more realistic estimate of the mortality burden for individuals with T2D.

### Model uncertainty

Probabilistic sensitivity analyses were conducted to assess the potential impact of uncertainty in key inputs on the simulation results. In these analyses, the uncertainty distributions of the prediction parameters were incorporated to simulate the development of T2D. We performed 100 simulations in a randomly selected 1% sample of the synthetic population, drawing from the uncertainty distribution of each input parameter. The resulting 95% uncertainty intervals were calculated based on the 2.5th and 97.5th percentiles of the 100 simulations.

### Model outputs

Throughout the simulation period, the model monitors and updates the levels of risk factors, as well as the T2D status and survival for each individual and model cycle. This includes the annual number of new and prevalent cases of each risk factor level and T2D, as well as the number of total deaths and those caused by T2D. The model provides mean estimated outcomes for the simulated population over the simulation period (2018–2030), including the prevalence and incidence of T2D for the adult population aged 35 years and older. Additionally, the burden was characterized by calculating DALYs, a time-based metric that combines the years of life lost (YLL) due to premature death and the years of healthy life lost due to disability (YLD), thus aggregating mortality and morbidity into a single statistic.

### Inequalities in the burden of T2D

The study also presented stratified results by age, sex, region, and education level. To quantify the gradient in T2D prevalence, incidence and DALY rates on both a relative and an absolute scale, two regression-based metrics of inequalities were used: the relative index of inequality (RII) and the slope index of inequality (SII) [22, 23]. RII and SII values greater than 1 and 0, respectively, would indicate wider inequality gaps between socio-demographic extremes. These metrics allow for a more nuanced understanding of the distribution of T2D burden among different groups within the population.

### Model validation

To ensure the internal validity of the population model, we compared the prevalence estimates of baseline characteristics, T2D, and associated risk factors from the synthetic population generated against the latest data from the BHIS and the BELHES in 2018, stratified by age, sex, and region. Additionally, the external validity of the T2D-M outputs was assessed by comparing the estimated number of new cases of T2D and those taking BP medication against the observed values from the Belgian Compulsory Health Insurance (BCHI) data for the years 2019 and 2020. Moreover, we used the latest BHIS and the BCHI data from 2018 to explore the agreement between self-reported and health administrative data for ascertaining the disease prevalence of T2D and hypertension.

## Results

### Baseline synthetic population characteristics

An overview of the baseline characteristics of the study synthetic population by age group is presented in Table 1. The prevalence of elevated BMI was 45.1%, elevated waist circumference was 37.3%, and high blood pressure was 18.5% in the population studied. These risk factors tended to be more notable in older groups. Similarly, the overall prevalence of hyperglycemia (5.0%) is also greater in older strata, and the prevalence of self-reported T2D ranged from 1.4% (ages 18–34) to 13.8% (ages 65+), with an overall prevalence of 6.2%. The similarity of the synthetic population generated and the corresponding (real) Belgian population of 2018 was evaluated as internal validation and intraclass coefficients (ICC) computed for the age-sex-province group on the prevalence of risk factors and T2D support the agreement between the T2D-M synthetic population and the nationally representative survey data (Additional file 1: Technical Annex).

**Table 1:** Descriptive characteristics (%) of the synthetic population of Belgium in the simulation baseline year 2018

		< 18 yrs (n = 2,301,495)	18–34 yrs (n = 2,374,218)	35–64 yrs (n = 4,561,902)	> 65 yrs (n = 2,138,454)	Overall (n = 11,376,069)
Sex	Men	51.17	50.27	50.09	44.04	49.21
	Women	48.83	49.73	49.91	55.96	50.79
Region	Brussels	11.94	12.93	10.07	7.37	10.54
	Flanders	55.25	54.95	58.19	61.83	57.60
	Wallonia	32.8	32.12	31.74	30.8	31.86
Nationality	Belgians	90.11	86.1	87.32	95.17	89.10
	Non-Belgians Europeans	5.94	6.51	7.65	4.06	6.39
	Non-Europeans	3.95	7.4	5.03	0.77	4.51
Education level	Low	12.21	12.16	14.08	38.44	17.88
	Intermediate	27.97	33.88	33.7	29.9	31.86
	High	59.82	53.96	52.22	31.66	50.26
Income level	Q1	4.98	7.07	9.61	20.93	10.27
	Q2	8.87	11.44	11.13	24.16	13.19
	Q3	16.7	16.73	18.75	23.5	18.81
	Q4	26.65	28.98	26.61	22.05	26.26
	Q5	42.8	35.76	33.89	9.36	31.47
Self-reported BMI status^1^	< 25	74.75	67.76	45.62	41.29	55.32
	25–30	14.97	22.98	35.78	38.86	29.84
	> 30	10.28	9.26	18.61	19.85	15.21
Measured waist circumference^2^	M: < 94 cm; F: < 80 cm	NA	62.02	34.83	18.49	38.1
	M: 94–102 cm; F: 80-88 cm	NA	20.94	26.8	24.18	24.6
	M: ≥ 102 cm; F: ≥ 88 cm	NA	17.04	38.37	57.33	37.3
High blood pressure^2^	Self-reported	NA	2.56	17.46	38.27	18.5
	Use of medication	NA	1.04	14.84	35.6	16.1
High blood glucose levels^2^	Blood glucose ≥ 126 mg/dl or HbA1c ≥ 6%	NA	1.34	3.7	11.95	5.0
Diabetes^2^	Self-reported	NA	1.37	5.07	13.83	6.2

^1^Based on self-reported weight and height; ^2^Based on measured values; ^3^Measured blood glucose of ≥ 126 mg/dL or measured HbA1c ≥ 6%

### Forecasting T2D incidence

**Fig. 1:**
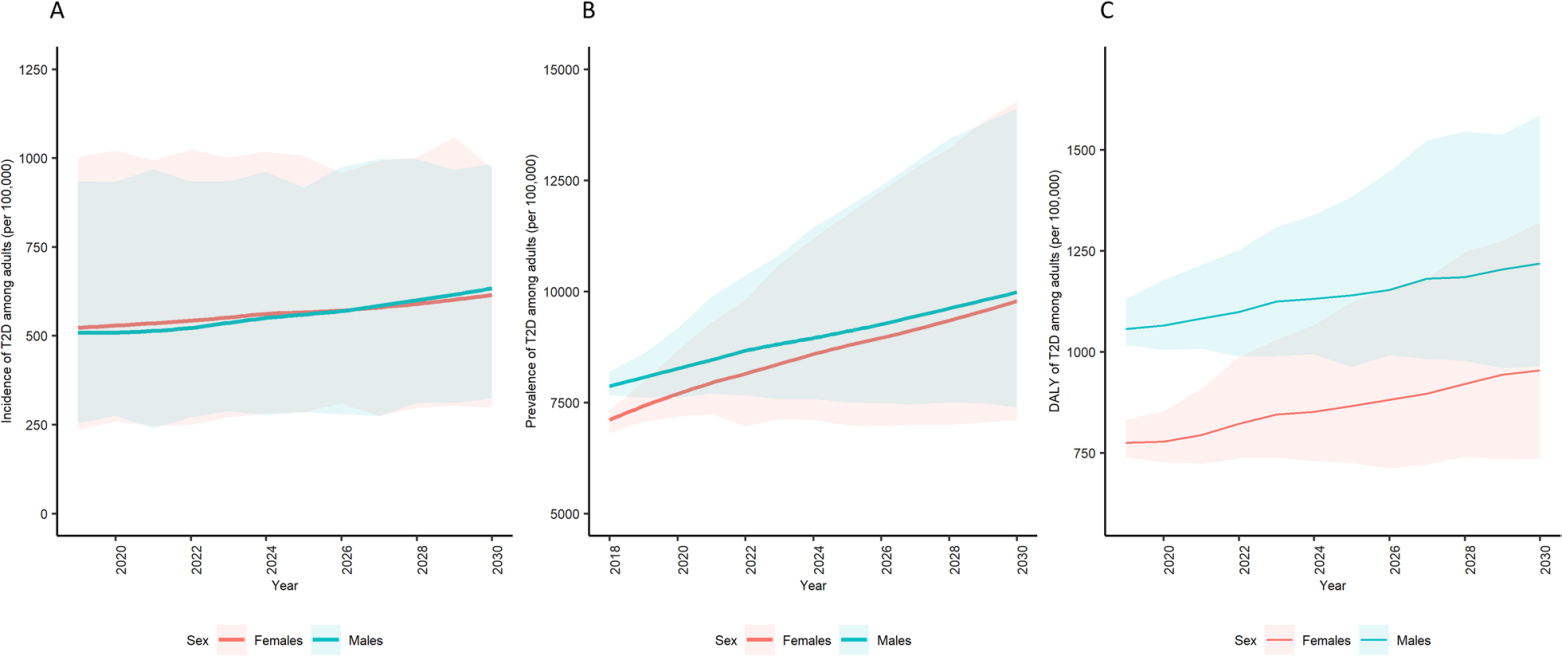
Forecast prevalence and incidence of type 2 diabetes in the adult Belgian population. **A** Incidence (2019–2030)**, B** prevalence (2018–2030), and **C** disability-adjusted life years (DALYs) (2019–2030)

**Fig. 2.**
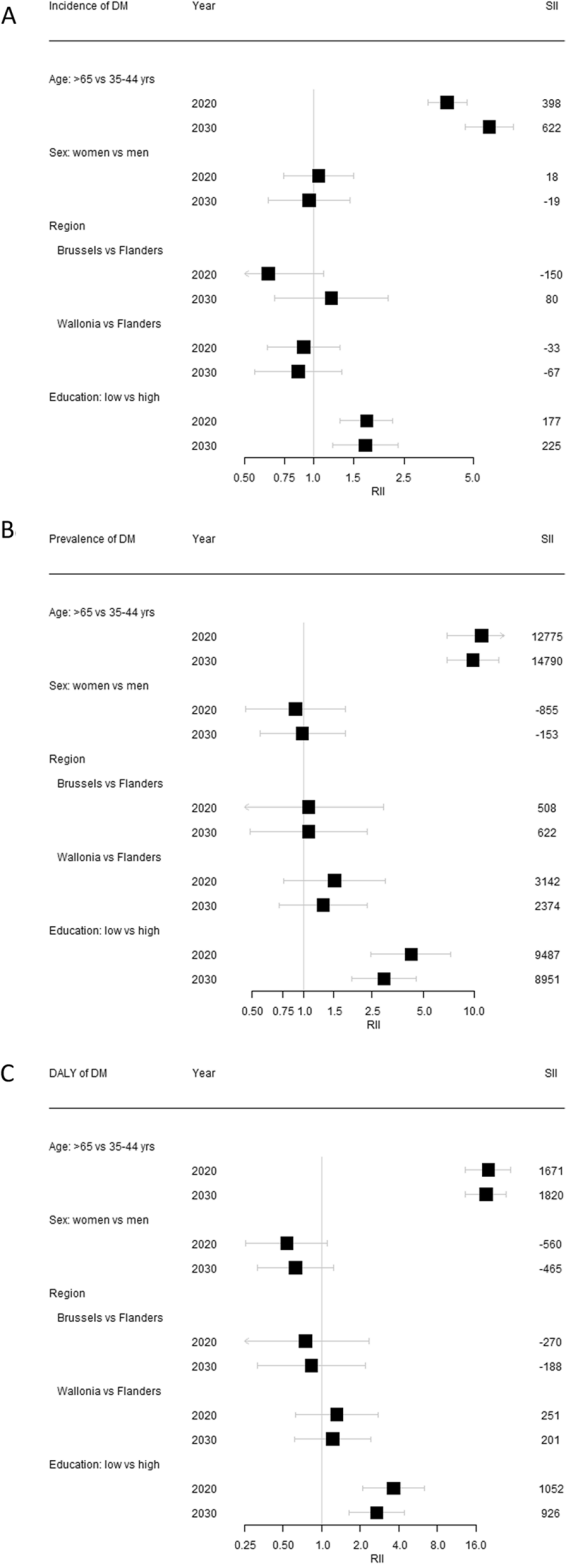
10-year changes (2020–2030) in disparities in the **A** incidence, **B** prevalence and **C** disability adjusted life years (DALYs) of type 2 diabetes in Belgium. *Abbreviations* RII, Relative inequality index; SII, slope inequality index. Footnote: Figure represents the quantification of the magnitude of disparities in the distribution of the burden of type 2 diabetes related to age (more than 65 years old versus 35–44), sex (women versus men), Belgian region (Brussels versus Flanders versus Wallonia), and Education (low versus high education)

As shown in Table 2**,** an average annual incidence of 560 [95% UI 285; 962] incident cases of T2D per 100,000 inhabitants aged 35–80 years are forecasted between 2019 and 2030 in Belgium, totalling 6722 [3421; 11,538] cases with a modest increase for the overall adult population without apparent differences based on sex (Fig. 1A). No substantial differences were observed across regions but average annual incidence rates appeared consistently higher for the group reporting lower levels of education (Table 2). These inequalities are also visible in Fig. 2A where the age- and education-related gap in T2D incidence was evident with an RII estimated as 5.1 [4.2; 6.3] for advancing age and 1.8 [1.4; 2.4] for low education.

**Table 2:** Forecast (2018–2030) of type 2 diabetes incidence, prevalence and DALY among adults, aged 35–80 years, in Belgium stratified by age, sex and region

Region	Sex	Education level	Incidence		Prevalence			DALYs		
			Average 2019–2030	Total 2019–2030	2018	2030	%change	2019	2030	%change
All	Both	All	560 (285; 962)	6722 (3421; 11,538)	7480 (7307; 7655)	9930 (7223; 14,187)	32.8	912 (588; 1454)	1088 (563; 2283)	19.3
	Females	All	558 (277; 990)	6698 (3328; 11,873)	7076 (6817; 7,312)	9784 (7104; 14,261)	38.3	775 (467; 1276)	954 (471; 2121)	23.1
	Males	All	555 (291; 946)	6668 (3500; 11,349)	7908 (7666; 8190)	9955 (7393; 14,111)	25.9	1056 (691; 1641)	1219 (653; 2448)	15.4
Brussels	Both	All	568 (321; 992)	6868 (3880; 11,971)	7667 (6907; 8286)	97,76 (7377; 13,921)	27.5	792 (420; 1362)	963 (448; 2113)	21.6
	Females	All	608 (331; 1043)	7351 (4005; 12,574)	7477 (6608; 8443)	10,284 (7582; 14,429)	37.5	707 (354; 1277)	883 (394; 2068)	24.9
		High	397 (211; 668)	4749 (2567; 8027)	4287 (3490; 5,293)	6806 (4698; 9171)	58.8	463 (208; 881)	608 (253; 1,384)	31.3
		Intermediate	732 (417; 1341)	8923 (5144; 16,353)	8265 (6194; 10,053)	11,968 (8992; 18,678)	44.8	731 (297; 1494)	1010 (429; 2563)	38.2
		Low	1233 (585; 2010)	14,830 (6993; 24,202)	16,771 (14,228; 19,818)	21,468 (14,650; 30,549)	28.0	1533 (730; 2901)	1856 (750; 4402)	21.1
	Males	All	570 (291: 956)	6843 (3,495; 11,469)	7832 (6764; 8,878)	9410 (7,017; 13,217)	20.1	875 (456; 1545)	1038 (501; 2134)	18.6
		High	479 (259; 801)	5,785 (3,122; 9663)	4,958 (4,015; 6,151)	6520 (4,561; 9,724)	31.5	684 (339; 1257)	767 (335; 1614)	12.1
		Intermediate	574 (323; 1097)	6992 (3904; 13,249)	10,176 (7,699; 12,518)	10,968 (8,240; 16,238)	7.8	934 (410; 1814)	1171 (545; 2550)	25.4
		Low	702 (302; 1189)	8520 (3655; 14,375)	12,959 (10,431; 15,667)	15,219 (12,000; 20,037)	17.4	1385 (641; 2585)	1581 (737; 3245)	14.2
Flanders	Both	All	552 (278; 940)	6627 (3332; 11,277)	6,863 (6,609; 7,111)	9,514 (6,814; 13,507)	38.6	892 (583; 1405)	1074 (549; 2205)	20.4
	Females	All	539 (296; 931)	6511 (3583; 11,233)	6,273 (5,952; 6,519)	8,986 (6,380; 13,084)	43.2	721 (440; 1197)	893 (434; 1965)	23.9
		High	470 (232; 826)	5655 (2782; 9924)	3,179 (2,779; 3,510)	6,817 (4,470; 10,337)	114.5	471 (269; 832)	681 (333; 1560)	44.6
		Intermediate	547 (269; 978)	6571 (3228; 11,731)	5,795 (5,199; 6,372)	9,562 (6,546; 14,035)	65.0	686 (397; 1169)	973 (442; 2150)	41.8
		Low	771 (346; 1344)	9230 (4037; 16,123)	15,021 (14,062; 16,154)	17,925 (13,667; 24,381)	19.3	1496 (834; 2509)	1741 (848; 3725)	16.4
	Males	All	599 (290; 1092)	7193 (3487; 13,102)	7,480 (7,084; 7,837)	9,998 (7,246; 14,033)	33.7	1064 (704; 1638)	1253 (663; 2490)	17.8
		High	516 (273; 835)	6213 (3280; 10,038)	4,763 (4,300; 5,151)	8,064 (5,505; 11,608)	69.3	774 (497; 1189)	1041 (535; 2058)	34.5
		Intermediate	579 (292; 994)	6948 (3510; 11,924)	9,896 (9,241; 10,622)	11,933 (9007; 16,318)	20.6	1299 (783; 2119)	1467 (759; 2859)	12.9
		Low	759 (367; 1394)	9040 (4365; 16,778)	9,861 (9,038; 11,109)	12,508 (8277; 19,542)	26.8	1402 (824; 2293)	1558 (767; 3405)	11.1
Wallonia	Both	All	564 (286; 1025)	6768 (3431; 12,306)	8,595 (8,303; 8,950)	10,742 (7,930; 15,573)	25.0	991 (610; 1600)	1149 (613; 2490)	15.9
	Females	All	537 (261; 924)	6446 (3136; 11,086)	8,427 (7,976; 8,898)	11,158 (8,239; 16,363)	32.4	890 (506; 1487)	1087 (549; 2450)	22.1
		High	472 (217; 900)	5659 (2602; 10,797)	5,178 (4,616; 5,786)	8,425 (5,958; 13,055)	62.7	573 (317; 995)	837 (414; 1932)	46.1
		Intermediate	644 (362; 1167)	7739 (4338; 13,995)	8,723 (7,636; 9,517)	12,635 (9,625; 17,819)	44.8	921 (500; 1606)	1210 (597; 2726)	31.4
		Low	813 (372; 1470)	9727 (4442; 17,614)	14,997 (13,872; 16,626)	17,053 (12,445; 23,975)	13.7	1568 (836; 2714)	1619 (726; 3599)	3.3
	Males	All	545 (279; 943)	6542 (3352; 11,313)	8,764 (8,215; 9,156)	10,098 (7571; 14,668)	15.2	1101 (682; 1746)	1215 (673; 2554)	10.4
		High	540 (274; 961)	6486 (3289; 11,530)	4,801 (4,195; 5,354)	8,055 (5,347; 12,906)	67.8	721 (420; 1170)	996 (526; 2,314)	38.1
		Intermediate	512 (256; 882)	6140 (3066; 10,562)	12,566 (11,351; 13,782)	12,197 (9709; 15,932)	-2.9	1429 (805; 2437)	1371 (731; 2692)	-4.1
		Low	608 (299; 1035)	7265 (3583; 12,455)	11,171 (9,797; 12,812)	11,741 (8767; 16,849)	5.1	1425 (821; 2396)	1440 (748; 2936)	1.1

### Forecasting T2D prevalence

The proportion of people living with T2D is expected to increase by 32.8% between 2018 and 2030, reaching 9930 [7223; 13,330]/100,000 cases for ages 35–80 years (Table 2). Prevalence rates are expected to remain comparable between sexes, with levels up to 9955 [7393; 14,111]/100,000 for men and 9784 [7104; 14,261]/100,000 for women in 2030 (Fig. 1B). While the region is not a significant source of inequalities in T2D overall, age and education are expected to be sources of disparity, with a widening gap in T2D prevalence foreseen up to 2030 (Fig. 2B). The lower education group is expected to have 15,161 [11,203; 21,290]/100,000 T2D cases in 2030 compared to 7568 [5285; 11,236]/100,000 among the highly educated (data not shown), corresponding to an RII of 2.9 (95% CI 1.9; 4.5) anticipated to narrow over the relevant period (p-trend = 0.017) across all regions of Belgium, as larger increases in prevalent T2D are expected among the highly educated (Table 2).

### Forecast of DALYs

The rate of DALYs related to T2D is expected to increase [+ 19.3%] for the simulated period from 2019 levels (912 [95% UI 588; 1454]/100,000), and hereby reaching levels of 1088 [563; 2,283]/100,000 in 2030 for the ages 35–80 years (Table 2). DALYs rates of T2D were expected to likely remain higher for men compared to women, with levels up to 1219 [653; 2,448]/100,000 for men and 954 [471; 1,221]/100,000 for women in 2030 (Fig. 1C), albeit greater increases over the simulation period are observed for women, and particularly among those of higher education (e.g. 46.1% for women in the Wallonia region). Likewise, to incidence and prevalence figures, an expected age-related and education-related gap in T2D DALY rates was foreseen to continue over the time course up to 2030 (Fig. 2C).

### Model external validation

The prevalence of T2D was validated using the BHIS2018 linkage with BCHI data from 2018 to 2020. Stratified by age, sex, and province, the ICC, as a measure of agreement, provided overall evidence that the T2D-Mis capable of modelling the prevalence of T2D-M for the first two years (years 2018–2020 with existing data) with good reliability (ICC 0.791 (95% CI 0.709, 0.851). Detailed information on the model validation is provided in the Additional file 1: Technical Annex.

## Discussion

This is the first study to forecast the T2D prevalence and incidence up to 2030 in Belgium, also providing an estimation of the burden of T2D, in terms of mortality and morbidity combined, and the evolution of existing inequalities between socio-demographic strata. The demographic characteristics, prevalence of risk factors and self-reported diabetes in the synthetic population generated mirrored those reported for the Belgian population in 2018 [13]. From this, we estimated that 6722 new cases are expected in the period 2018–2030 with the greatest proportion of cases observed among advanced age groups, and the lower education subgroups. This high prevalence of T2D in Belgium comes along with an increased number of people living with T2D-related disabilities which has been observed in the increasing number of DALYs through the simulation period. This is aligned with the figures projected for the European region, where the number of people living with diabetes is expected to rise to 67 million by 2030 [3], and to 69 million by 2045 [2] from the current 61 million.

In Europe, geographical disparities in the distribution of T2D exist, and there is also considerable variation within countries, particularly related to socioeconomic strata [24] and minorities [25]. Socioeconomic inequalities in T2D prevalence are likely due to differences in exposure to lifestyle-related risk factors such as unhealthy diets and physical inactivity, which lead to metabolic risk factors such as elevated BMI, hypertension, and hyperglycemia [26, 27]. While variables such as age, sex, geographical region, and socioeconomic status are well-established indicators of health disparities at the population level, as demonstrated in the EU [28], our previous work on the trends from 1997 to 2018 highlighted significant avoidable disparities between extremes based on socioeconomic status, as defined by education and income strata in Belgium. This disparity was particularly evident in lifestyle-related risk factors, high BMI prevalence, and consequently T2D [29]. The high prevalence of lifestyle- and metabolic-related risk factors, coupled with an aging population, is contributing to the increasing number of people with T2D [4]. In Belgium, the reported prevalence of T2D is higher in the Walloon region and the Brussels Capital region compared to the Flemish region [30], which is also apparent in our simulation. The prevalence is also higher for individuals with a lower socioeconomic status. These socioeconomic differences in the prevalence of diabetes are likely exacerbated by the prevalence of unknown or insufficiently controlled diabetes [30].

According to the Official information and services of Belgium, 6.6% of the Belgian residents had a known diabetes diagnosis in 2020 as assessed by the IMA-AIM Atlas [31]. However, the true prevalence is estimated at 10% accounting for a large number, approximately one in three people with diabetes, of undiagnosed cases [4]. Following the increasing time trend in T2D, our simulation forecasts an increase in the prevalence of excess weight, waist circumference, high blood pressure, and particularly hyperglycaemia. Factors contributing to this increase include an aging population, highly prevalent sedentary lifestyles, and unhealthy eating habits [4].

Microsimulation models are used by policymakers to evaluate policy impacts on different population groups. In population health, this model can estimate the effects of strategies and health policies on the T2D burden in Belgium. For instance, it can analyse the impacts of fiscal policies on target foods, education programs, or community-based interventions on the overall population and specific groups. Microsimulation models provide insights into the distributional impacts of policy proposals, allowing policymakers to create more effective and equitable policies. However, these models are only as reliable as their assumptions and data inputs, so careful consideration and validation of these factors are necessary to ensure accuracy. The T2D-M operates under several key assumptions to simulate transitions in risk factors, T2D status, and survival over time. Firstly, individual weights are utilized for population generation, assuming a household size of one and independence among individuals despite clustering in the sampling process. The model structure involves an open cohort design incorporating births and deaths while omitting migration, or information of non-Belgian residents due to data constraints which likely underestimates T2D burden. Further, socio-economic status could only be approached through levels of income and education which might not capture completely the nuances of these determinants. T2D onset is not simulated for children, and T2D risk estimation begins from age 35, as informed by the FINDRISC equation. Survival status is updated considering age-sex-specific relative risk estimates for individuals with diabetes. Model uncertainty is primarily attributed to parameter variations in the T2D risk prediction equation, with relatively minor uncertainty in transition probabilities and mortality rates due to narrow confidence intervals and extensive observed data coverage. External validation compares prevalence estimates of hypertension from self-reports and administrative data, while higher mortality rates for T2D individuals are linked to changes in risk factor prevalence driving disease incidence and mortality. These assumptions should collectively inform the model's limitations and interpretation of results.

In future developments it would be important to understand the comparative contribution of aging and modifiable risk factors in the predicted T2D prevalence for prioritization of strategies. Further, more granular age groups will provide further insight into differential burdens. It is also important to note that the predictions presented here are subject to change based on various factors, including changes in public health policies and advances in T2D prevention and treatment. Our model forecast a modest reduction in the gap in the burden of T2D between socioeconomic groups due, mostly, to a steep increase in the prevalence burden among the higher-education subgroups across the three regions in Belgium. While this may reflect demographic transitions into extended life expectancy, it still calls for the strengthening of preventive measures across all socioeconomic groups to reduce the burden overall and reach equitable health.

## Conclusions

This increasing burden of T2D is concerning and highlights the urgent need for effective prevention and management strategies to address this growing epidemic. Accurately monitoring risks and disease prevalence systems in the population is essential for effective public health planning. By analysing trends and identifying health disparities, we can better understand which groups are most affected and develop targeted interventions to improve their health outcomes. Utilizing a model that incorporates age, sex, region, and education-specific transition probabilities between risk factors levels and health states, such as the T2D-M in this study, we are better equipped to study future projections of these health disparities. This approach enables us to identify groups that may be at higher risk for the further development of targeted interventions to address these disparities and improve overall population health.

### Supplementary Information

**Additional file 1**. Technical Annex.

## Data Availability

The source data that support the findings of this study are publicly available from Sciensano, and the Belgian statistical office (STATBEL). Linkage of specific databases (BHIS and BCHI) for validation purposes were used under license for the current study, and permission should be obtained from the Intermutualistic Agency (IMA-AIM), and Sciensano.
