# Supplementary material for: The future burden of type 2 diabetes in Belgium: a microsimulation model
Source: Popul Health Metr. 2024 Apr 23;22:8. doi: 10.1186/s12963-024-00328-y (PMC11040979; doi:10.1186/s12963-024-00328-y)
Supplement: Supplementary file 1 — Additional file 1. Technical Annex. [file 12963_2024_328_MOESM1_ESM.docx]

**The future burden of type 2 diabetes in Belgium: a microsimulation model**

**Technical Annex**

[***1.***](#_heading=h.30j0zll) ***Summary 3***

[**1.1.**](#_heading=h.1fob9te) **Overall description 3**

[**1.2.**](#_heading=h.2et92p0) **Technical information 3**

[***2.***](#_heading=h.tyjcwt) ***Sources of input parameters 3***

[**2.1.**](#_heading=h.3dy6vkm) **Belgian Population**  **3**

[**2.2.**](#_heading=h.4d34og8) **Belgian Health Interview Survey (BHIS) 5**

[**2.3.**](#_heading=h.2s8eyo1) **Belgian Health Examination Survey (BELHES) 6**

[**2.4.**](#_heading=h.17dp8vu) **Standardised Procedures of Mortality Analyses (SPMA) 6**

[**2.5.**](#_heading=h.3rdcrjn) **Belgian Compulsory Health Insurance (BCHI) 6**

[**2.6.**](#_heading=h.26in1rg) **T2D risk prediction equation 6**

[**2.7.**](#_heading=h.lnxbz9) **Relative risks 7**

[***3.***](#_heading=h.35nkun2) ***Generation of the synthetic population 7***

[**3.1.**](#_heading=h.1ksv4uv) **Use of survey data for informing the synthetic population 7**

[3.1.1. Step 1: BELHES-2018 as donor data to recipient BHIS-2018 7](#_heading=h.44sinio)

[3.1.2. Step 2: BHIS-2018 complemented with BELHES-2018 7](#_heading=h.2jxsxqh)

[3.1.3. Step 3: BHIS 2018 as the survey micro-dataset for the child population 8](#_heading=h.z337ya)

[**3.2.**](#_heading=h.3j2qqm3) **Algorithm for generating the synthetic population 8**

[**3.3.**](#_heading=h.4i7ojhp) **Follow-up of the synthetic population 8**

[***4.***](#_heading=h.2xcytpi) ***Model structure 12***

[**4.1.**](#_heading=h.1ci93xb) **Age update 12**

[**4.2.**](#_heading=h.2bn6wsx) **Risk factors update 12**

[**4.3.**](#_heading=h.1pxezwc) **T2D status update 18**

[**4.4.**](#_heading=h.2p2csry) **Survival status update 19**

[***5.***](#_heading=h.3o7alnk) ***Model output 19***

[***6.***](#_heading=h.ihv636) ***Model uncertainty 20***

[**6.1.**](#_heading=h.32hioqz) **Individual heterogeneity 20**

[**6.2.**](#_heading=h.1hmsyys) **Structural uncertainty 20**

[**6.3.**](#_heading=h.2grqrue) **Stochastic uncertainty 21**

[**6.4.**](#_heading=h.vx1227) **Parameter uncertainty 21**

[***7.***](#_heading=h.4f1mdlm) ***Model validation 21***

[**7.1.**](#_heading=h.2u6wntf) **Validation of the synthetic population 21**

[**7.2.**](#_heading=h.19c6y18) **Validation of the self-reported data 22**

[**7.3.**](#_heading=h.28h4qwu) **Validation of model output 23**

[7.3.1. External validation: Prevalence of risk factors 23](#_heading=h.nmf14n)

[7.3.2. External validation: Prevalence and incidence of T2D 24](#_heading=h.37m2jsg)

[7.3.3. External validation: Mortality 24](#_heading=h.46r0co2)

[***8.***](#_heading=h.111kx3o) ***List of assumptions 26***

[***9.***](#_heading=h.3l18frh) ***References 27***

# Summary

## Overall description

The type 2 diabetes microsimulation model (T2D-M) is built as a discrete-event state-transition microsimulation with two possible events, incident type 2 diabetes (T2D) or death, updated annually over the simulation period (normally 10 years). The model recreates the Belgian population (all ages) into a synthetic (theoretical) population informed by the national-representative (age, sex and Belgian province) distributions of socio-economic factors (household income and household education level), and relevant risk factors for T2D (body mass index (BMI), waist circumference, use of blood pressure (BP) medication, and high blood glucose) as well as prevalent T2D. For each synthetic individual, represented by a unique identifier and a set of associated attributes at baseline, the model simulates probabilistic transitions through the life-course updating of attributes and risk factors as individuals age and predicting the occurrence of events up to death or the end of the simulation period. For this life-course simulation, in one-year intervals, each synthetic individual is subjected to the following set of subsequential stochastic rules: 1) update of risk factors using strata-specific transition probabilities after increasing age by one year, 2) update T2D status in healthy individuals using probabilities of developing T2D as estimated from the FINDRISC equation, and thereafter 3) update survival status using strata-specific probabilities of death. After all individual transitions are combined, the model’s output is an estimate of the incidence, prevalence, and disability-adjusted life years (DALYs) of T2D in the synthetic population, incorporating all changes in T2D risk factors, in particular excess weight. **Figure 1** provides a graphical representation of the model structure. In the following sections a description of the methodologies for each of the steps in the T2D-M, including the sources of the input parameters and their main assumptions and application in the model, is provided.

## Technical information

T2D-M is programed in R v4.1.2 and is currently deployed on a High-Performance Computing cluster, i.e., a NEC system consisting of 152 computing nodes with two 32-core AMD Epyc 7452 CPUs and 256 GB RAM each hosted by the Vlaams Supercomputer Centrum (VSC) (1). Relevant R packages during model development were: *mice* responsible for handling missing survey data (2); *SimPop* for generating the synthetic population (3); *lpSolve* (4), *mgcv* (5), and *demography* (6) for estimating transition probabilities for risk factor states following the simplex algorithm optimisation, and death, respectively; and the R package *foreach* (7) used for parallel computation in the VSC computing cluster.

# Sources of input parameters

The T2D-M is built upon nationally representative databases of Belgium, and published risk equations and risk ratios used in the different stages of the model, as detailed In **Table 1** and **Figure 1**.

## Belgian population

The Belgian Register , as published yearly by the Belgian Statistical Office (STATBEL), provides population statistics of the residential population, as recorded in the National Register of Natural Persons on January 1 of the reference period in question (8). Population statistics include the number of residents in the country, including Belgian nationals and non-Belgian inhabitants admitted or authorized to settle or stay on the territory, and their associated demographic variables of age, sex, place of residence, civil status, nationality, and household’s structure. In addition, STATBEL in collaboration with Federaal Planbureau, provides also historical and forecasted metrics on determinants of population growth namely mortality, fertility, and internal and international migration for the years 1992-2070, with forecasts from 2021 onwards calculated based on historical trends (8). From these sources, information on the population structure by age, sex and province for the year 2018 was retrieved, matching the year of the latest survey representative of the Belgian population. Further, information on the number of births by age, sex and province for the simulation years was retrieved to afford a dynamic synthetic population.

**Figure 1** Visual representation of the T2D-M, a discrete-event state-transition microsimulation model


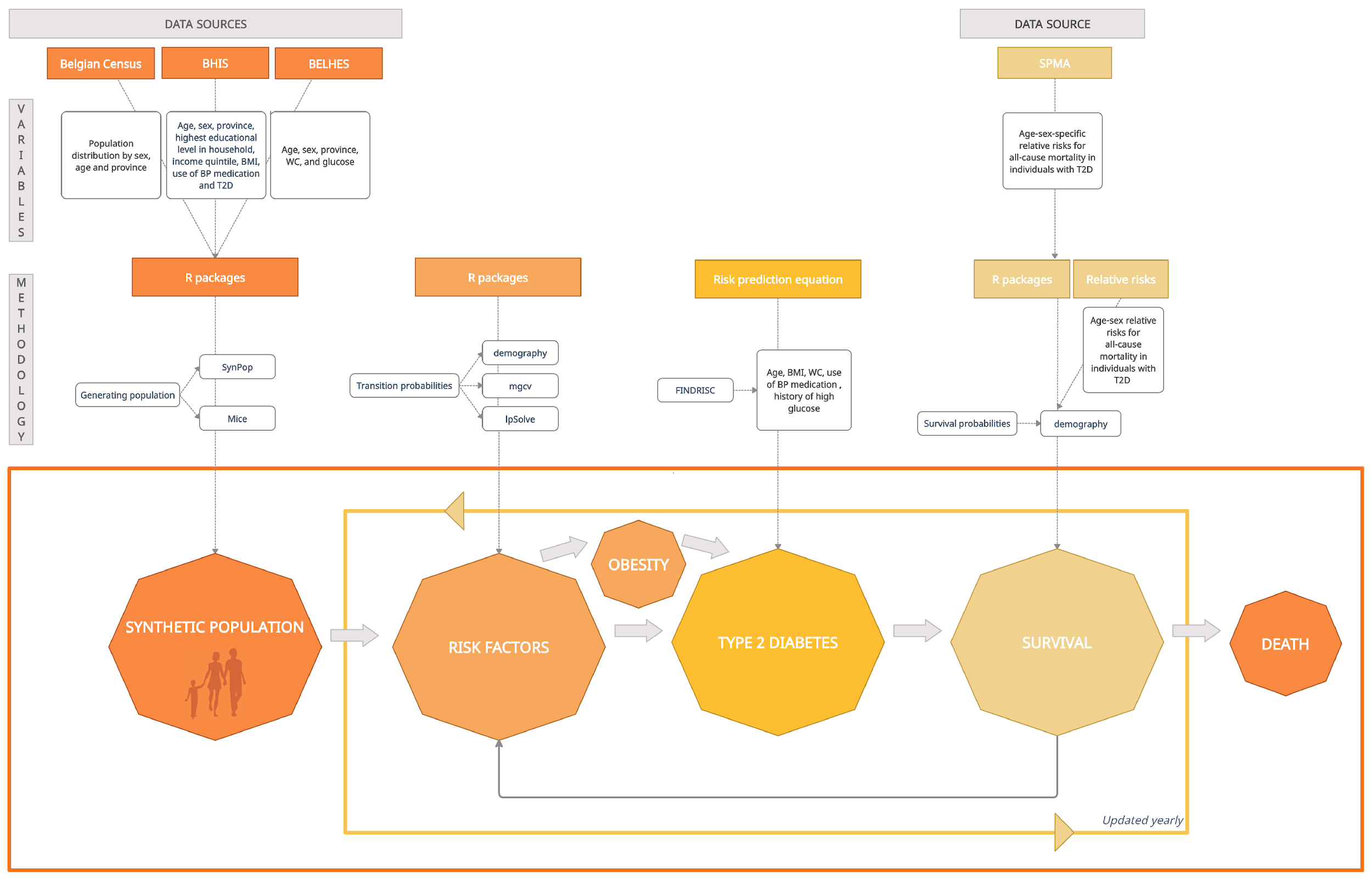


**Table 1** Input data sources, and variables used for the development and validation of the T2D-M

| **Input data sources** | **Brief description** | **Variables included in T2D-M** | **Use in T2D-M** |
| --- | --- | --- | --- |
| Belgian Population (8) | Residential population count, carried out annually. Population projections | Population distribution and projections by age, sex and province for the year 2018 and the forthcoming years of simulation | - Generation of synthetic population - Population follow-up |
| Belgian Health Interview Surveys (BHIS) (9) | Cross-sectional survey, carried out periodically every four to five years since 1997, including a sample of ~10,000 participants per survey wave representative of Belgian residents | Age, sex, province, highest educational level in household, household income quintile, self-reported risk factors (BMI, use of BP medication) and self-reported prevalent T2D for the year 2018 | - Generation of synthetic population - Estimation strata-specific risk factor transition probabilities - Internal validation |
| Belgian Health Examination Surveys (BELHES) (10) | Cross-sectional survey, carried out for the first time in 2018, as a subsample of ~1,000 BHIS adult participants representative of Belgian residents | Age, sex, province, measured waist circumference, and measured blood glucose levels for the year 2018*^1^* | - Generation of synthetic population - Estimation of strata-specific risk factor transition probabilities - Internal validation |
| Standardised Procedures of Mortality Analyses (SPMA) (11) | Belgian vital statistics, collected annually from 1987 onwards | Age group, sex, and province all-cause and diabetes-specific mortality rates for all available years, and proportional mortality rate from diabetes | - Estimation of strata-specific one-year probability of death |
| Belgian Compulsory Health Insurance (BCHI) data (12) | Individual-level patient data from the health insurance funds with linkage to BHIS 2013, and 2018 | Prevalent chronic conditions defined by reviewing prescription records using the Anatomical Therapeutical Chemical (ATC) / Defined Daily Dose (DDD) Index for the year 2018, 2019 and 2020*^1^* | - External validity |
| Finnish Diabetes Risk Score (FINDRISC) (13) | Risk prediction equation to identify individuals at high risk for T2D in the next 10-years | Concise model including age, BMI, waist circumference, use of BP medication and history of high blood glucose as predictors*^1^* | - Estimation of one-year probability of developing T2D |
| Relative risks | Relative risks (RR) for all-cause mortality in individuals with T2D (14) | Age-sex-specific RR*^1^* | - Inflation of the age-sex-province-specific one-year probability of death for individuals with T2D |

*^1^ Only available or relevant for the adult population, aged 18 years and older*

## Belgian Health Interview Survey (BHIS)

The BHIS is a cross-sectional survey, conducted by Sciensano since 1997, and carried out periodically every four to five years. This nationwide survey includes a sample of approximately 10,000 participants of all ages per survey wave, representative of registered residents in Belgium in terms of age, sex and province of residence (9). Details of the survey design are described in detail elsewhere (9, 15). Briefly, participants are selected from the Belgian Register through a multistage stratified population sampling involving a geographical stratification according to the three Belgian regions, and subsequently, a selection of municipalities within the ten provinces of Belgium, and finally households within municipalities, allowing for a maximum of four respondents within households. Survey questionnaires are administered face-to-face at the participant’s household retrieving self-reported information on demographics, specific diseases and conditions, and nutritional status. Information on health behaviors and lifestyle factors, including self-reported weight and height, was collected through self-administered questionnaires. Survey weights were calculated and applied to ensure the representativeness of the results originating from the population sample. The BHIS was authorized by an independent administrative authority protecting privacy and personal data, and was approved by the ethical committee of Ghent University Hospital. Information of the latest (2018) BHIS from 2,172 participants aged 0-17 years old and 9,439 participants aged 18 years and older was retrieved for generating the synthetic adult population, so that each synthetic individual has a minimum set of associated attributes of demographics, prevalent self-reported risk factors relevant for predicting T2D (i.e., body mass index (BMI), and, for adults, the use of (blood pressure) BP medication) and, for adults, prevalent self-reported T2D status, all needed to simulate his/her life course. Furthermore, estimates on the prevalence of self-reported risk factors by age, sex, region, and education level, as available from all BHIS survey cycles (1997, 2001, 2004, 2008, 2013 and 2018), were used to calculate the strata-specific transition probabilities for a change in risk factor state, including their corresponding time-trends for the prevalence of self-reported risk factors.

## Belgian Health Examination Survey (BELHES)

The BELHES is a cross-sectional survey study complementary to the BHIS. Developed by Sciensano, and carried out for the first time in 2018, the survey includes approximately a subsample of 1,000 BHIS participants, aged 18 years and older, representative of Belgian residents in terms of age, sex and province of residence (10). Details of the survey design are described elsewhere (10, 16). Briefly, in a subset of eligible adult BHIS participants additional health data were collected at the participants’ home by a trained nurse through physical examination and a sample of blood and urine as well as questions addressing circumstances of the measurements, inclusion/exclusion criteria for blood and urine collection, including medication use. Physical examinations included anthropometrics (height and weight to calculate BMI, and waist circumference) and blood pressure (systolic (SBP) and diastolic (DBP)). Laboratory analyses from a single blood draw included fasting glycemia and hemoglobin A1c (HbA1c) as well as fasting total serum (TC), high density lipoprotein (HDL) and non-HDL-cholesterol, and triglycerides (TG). Information from the 1,184 participants aged 18 years and older available from the BELHES-2018 was retrieved for complementing the self-reported information collected in BHIS 2018 in order to generate the synthetic population, in particular information on waist circumference and blood glucose, which are key inputs to the T2D-M. As done for the BHIS, the strata-specific risk factor transition probabilities for the relevant measured risk factors were calculated using estimates on the prevalence of risk factors by age, sex, region and education from the BELHES.

## Standardized Procedures of Mortality Analyses (SPMA)

The SPMA databases are developed by Sciensano and provide vital statistics since the early 1990s, with the latest release in 2019 (11). Cause-specific deaths were coded using ICD-9 for the years 1987-1997 and the ICD-10 from 1998 onwards using the initial cause of death and are available for the total population as well as stratified by age, sex, region, province of residence and nationality. For the T2D-M, data on all-cause mortality and T2D-specific mortality (ICD-10: E11-E14), grouped by age, sex and province, were retrieved for estimating one-year probabilities of death.

## Belgian Compulsory Health Insurance (BCHI)

The BCHI data, managed at the InterMutualistic Agency (IMA), gathers patient data from the health insurance funds with the aim to maintain and improve the performance, quality and accessibility of the Belgian health care and health insurance systems (12). Such data include patient-specific information on the medicines and medical devices that are dispensed in pharmacies as part of the process for reimbursement of the compulsory health insurance in Belgium. It includes information on patient s’ identifiers , date of dispensing, and medicine identified using the Anatomical Therapeutical Chemical (ATC) Classification code and Defined Daily Dose (DDD). The ATC/DDD index was used to identify chronic patients defining a consumption of at least 90 DDD of chronic-indicated medication as indicative of a treated chronic condition (17). By linking BCHI and BHIS data using unique patient’s identifiers, the rates of prevalence of hypertension, hypercholesterolemia and diabetes in Belgium for the year 2018, 2019 and 2020, obtained from the two different sources could be compared and used for external validation of the synthetic population (2018) and the two subsequent model updates (2019, and 2020).

## T2D risk prediction equation

The Finnish Diabetes Risk Score (FINDRISC) is a risk prediction tool to quantify the 10-year risk of incident T2D, and was developed in the FINRISK Studies, a prospective cohort of Finns aged 35-64 years (13) and externally validated in different population settings, including Belgium (18). The original concise model is based on a multivariate logistic regression model including age, BMI, waist circumference, use of BP medication and history of high blood glucose (non-invasive) as predictors of T2D risk. The FINDRISC equation was used to estimate, for each synthetic individual, aged at least 18 years, the probability of developing T2D throughout the simulation period. Hereby, no risk of T2D is assumed for children and adolescents (see heading 8, list of assumptions), and for adults, it is assumed that age does not play a role in T2D risk until 45 years old.

## Relative risks

The estimations of age, sex, and province-specific one-year probability of death for individuals with T2D in Belgium were further adjusted with estimations from the Diabetes Epidemiology: Collaborative analysis of Diagnostic criteria in Europe (DECODE) study (14), a large population-based cohort in European populations, to obtain a more realistic estimate of the mortality burden for individuals with T2D.

# Generation of the synthetic population

The generation of the synthetic population is based on the National Register data for the reference population structure needed to produce margins of relevant categorical socio-demographic variables of age, sex, and province. Further, the survey data are used as inputs for generating a close-to-reality synthetic population following the algorithm implemented in the R package *SimPop* (3).

## Use of survey data for informing the synthetic population

The BHIS-2018 was the basis to extract the variables necessary for the generation of the synthetic population (all ages). Next, the subsample BELHES-2018 (18+ years old) was used to inform the synthetic population with objectively measured risk factors needed for the utilisation of FINDRISC affording fully informed survey data to be used in *SimPop* (3).

### Step 1: BELHES-2018 as donor data to recipient BHIS-2018

In the T2D-M, of the relevant variables to calculate the risk of incident T2D according to the concise FINDRISC, age, BMI and use of BP medication were available in BHIS, but waist circumference and history of high blood glucose (as objectively measured by either fasting plasma blood glucose or HbA1c) was collected in BELHES only. Because of the relatively small sample size of BELHES, it was necessary to generate these variables for all BHIS participants using a regression-based approach with BELHES serving as donor data and BHIS as recipient data (3, 19). For the regression models, a list of variables considered relevant and available in BHIS was pre-selected in order to generate the logarithm of waist circumference (i.e. age, sex, province, educational level and BMI), and fasting plasma blood glucose and HbA1c (i.e. additionally including T2D status, and a variable indicating currently dieting and/or taking medications). Unavailable data in the BELHES survey were handled by ten-fold multiple imputation by chained equations (MICE) from the R package *mice* (2). Univariate imputation model of each incomplete variable was specified as linear regression for numerical variables, logistic regression for two-level categorical variables and multinomial logistic regression model for multiple-level categorical variables with predictors selected based on the minimum proportion of usable cases of 25% and an absolute correction with the target variable or with the response indicator of at least 20%, and ever including age, sex and province (**Table 2**), and with the variables visiting in increasing order of the number of missing data. This MICE-approach implies that the regression models were fitted for each imputed dataset for a total of ten intermediate datasets.

### Step 2: BHIS-2018 complemented with BELHES-2018

The model coefficients, obtained from the fitted regressions in the BELHES, are used to predict the values for those variables for all the individuals of the BHIS data. To avoid all individuals with the same variable-mix for the independent variables having the same predicted value, a distinct random value was added to the predicted value for each individual. These random values (or error terms) were drawn from the distribution of the residuals of the prediction models. In our case, obtaining the error from a normal distribution of the residuals was preferred because each individual would have his/her own distinct error values, as illustrated in Templ et al. (3). The normal distribution was fitted with the median of the residuals as the mean and the absolute median deviation around the median (MAD) as the variance, and in this way non-sensitive to outliers and not dependent on sample size (20). The predicted value from the regression model added with a distinct error term for each individual becomes the predicted value for the particular continuous variable of interest, i.e. waist circumference, and fasting plasma blood glucose and HbA1c. Similar to the BELHES survey, missing data in the BHIS were handled by ten-fold MICE (2), and following a similar approach for imputation (**Table 3**). The ten imputed datasets were stacked together to create a large dataset, and this dataset with the stacked multiple imputation was the recipient data for the additional variables as collected by BELHES. As missing data of BELHES were also handled by MICE, the additional variables were generated for each set of the regression coefficients, as obtained from the regression models fitted for each imputed dataset of BELHES, and subsequently the predicted values were pooled following Rubin’s rule to have one predicted value for each individual in the dataset of BHIS with the stacked multiple imputation. This dataset with the stacked multiple imputation including original variables as collected by the BHIS and complemented with additional variables from BELHES allows for generating a fully informed adult synthetic population after dividing the survey weights by ten, the number of imputed datasets.

### Step 3: BHIS 2018 as the survey micro-dataset for the child population

Similar to the survey micro-dataset for the adult population, missing data on the child population in the BHIS 2018 were handled by ten-fold MICE using the R package *mice* (2), and following a similar approach for imputation (**Table 4**). The ten imputed datasets were stacked together to create a large dataset and afford generating a fully informed child synthetic population after dividing the survey weights by ten, the number of imputed datasets. In addition, with regard to the variable BMI in this population, the child growth standard BMI-for-age with associated z-scores taken from the WHO Growth Standards (21, 22) were used to stratify the child population according to BMI status, and hence measure prevalence estimates of childhood overweight/obesity.

## Algorithm for generating the synthetic population

Using the fully-informed Belgian survey data from 2018, (i.e. the stacked imputed BHIS, and for adults complemented with additional BELHES variables), a synthetic population was generated with a ‘close-to-reality’ distribution of the Belgian population structure of 2018 using the model-based algorithm implemented in R package S*imPop* (3, 19). A detailed description of this procedure has been previously reported (3). Briefly, the individual level data from the representative survey and the country-level demographics (National Register data) were taken as inputs to generate population level data in stages. Individual sampling weights of the survey were recalibrated using the generalized raking procedure to match the aggregate totals from the country’s population structure of 2018 by age, sex and Belgian province. Further, the household size in each stratum (province) was estimated and then using a weighted sampling, households were sampled (carrying their respective basic variables, such as age and sex distribution) in each stratum (province) to match the marginal totals. Finally, categorical variables and continuous variables were estimated. In our implementation, household weights were not available from BHIS, and hence the synthetic population was generated under the assumption of a household of size one, implying all individuals are independent and used the available individual weights instead of household weights (see heading 8, list of assumptions).

To subsequently generate the categorical and continuous variables, all relevant variables as basic household variables were included and used in the sampling, so that all of them were represented (instead of only age and sex), and therefore including also province, nationality, education level, income quintile group, BMI, waist circumference, use of BP medication, history of high blood glucose, other major lifestyle variables (alcohol consumption distribution, smoking status, physical activity level, and diet; consumption frequencies of fruit, vegetables, sugar-sweetened beverages and snacking) and T2D status. This allowed us to maintain the correlation structure between these variables that could have been missed in the modeling approach used in *SimPop*.

## Follow-up of the synthetic population

In the current implementation, the synthetic population is followed-up as an open cohort of individuals (all ages) from 2018 until 2030, monitoring the transitions of risk factors and the occurrence of events. In each annual cycle, the model incorporates newborns based on the number of births per year as reported by the Belgian national population projections for sex and province (23). Information on newborns was incorporated by bootstrapping existing model individuals aged 0-2 years of the same sex and province in order to maintain the covariance of demographic, anthropometric and major lifestyle-related characteristics. ‘New-migrated’ synthetic individuals, however, cannot enter the simulation after initiation, nor ‘existing’ synthetic individuals leave due to migration, but synthetic individuals will however leave the microsimulation when they die or the simulation period ends (see heading 8, list of assumptions).

**Table 2** Missing (%) values in selected variables from BELHES-2018, and predictors selected for imputation using the Predictor Matrix function of the R package *mice*

| **Variable** | **Variable type** | **Missing (%)** | **Additional variables, other than age, sex, and province, to inform imputation** |
| --- | --- | --- | --- |
| Nationality | Categorical (3 levels) | 1 (0.08%) | - |
| Household education level | Categorical (4 levels) | 22 (1.9%) | in, ltpa, hba1c |
| Income quintile | Categorical (5 levels) | 103 (8.7%) | edu, ltpa, height |
| Frequency of fruit consumption | Categorical (5 levels) | 1 (0.08%) | smk |
| Frequency of vegetables consumption | Categorical (5 levels) | 1 (0.08%) | - |
| Frequency of SSBs consumption | Categorical (5 levels) | 1 (0.08%) | smk, hdl |
| Frequency of snacking consumption | Categorical (5 levels) | 1 (0.08%) | - |
| Smoking status | Categorical (3 levels) | 80 (6.8%) | fr and ssbs |
| Distribution of alcohol consumption | Categorical (5 levels) | 94 (7.9%) | - |
| Leisure-time physical activity | Categorical (3 levels) | 114 (9.6%) | edu, in, height |
| Self-reported height | Continuous | 5 (0.4%) | in, ltpa, weight, waist, hdl |
| Self-reported weight | Continuous | 8 (0.7%) | height, waist, dbp, sbp, fpg |
| Self-reported BMI | Continuous | 11 (0.9%) | Calculated from imputed self-reported weight and height |
| Self-reported high cholesterol levels | Categorical (2 levels) | 3 (0.3%) | hbp, hbpm, hb1ac, tg |
| Self-reported high cholesterol levels and following a diet | Categorical (2 levels) | 3 (0.3%) | hbpd, dmd |
| Self-reported high cholesterol levels and taking medication | Categorical (2 levels) | 3 (0.3%) | hbp, hbpm, dm, dmm, waist, sbp, hba1c |
| Self-reported other serious heart disease | Categorical (2 levels) | 1 (0.08%) | MI, chd |
| Measured waist circumference | Continuous | 12 (1.0%) | edu, bmi, hbp, hbpm, dbp, sbp, fpg, hba1c, hdl, nonhdl, tg |
| Measured systolic blood pressure | Continuous | 5 (0.4%) | edu, weight, bmi, hbp, hbpd, hbpm, hclm, waist, fpg, hba1c, tg |
| Measured diastolic blood pressure | Continuous | 5 (0.4%) | edu, weight, bmi, hbp, hbpd, hbpm, waist, tg |
| Measured fasting plasma glucose | Continuous | 210 (18%) | edu, weight, bmi, dm, dmd, dmm, waist, sbp, hba1c |
| Measured haemoglobin A1c | Continuous | 119 (10%) | edu, bmi, hbp, hbpm, dm, dmd, dmm, hclm, waist, sbp |
| Measured total cholesterol plasma | Continuous | 189 (16%) | edu, bmi, hcl, hcld, hclm, tg |
| Measured HDL-cholesterol | Continuous | 187 (16%) | edu, ssbs, bmi, hcl, hcld, hclm, waist, tg |
| Measured non-HDL-cholesterol | Continuous | 187 (16%) | edu, bmi, hcl, hcld, hclm, waist, tg |
| Measured triglycerides | Continuous | 177 (15%) | edu, weight, bmi, hcl, hcld, hclm, waist, dbp, sbp, tc, hdl, nonhdl |

*Abbreviations: alc, distribution of alcohol consumption; chd, self-reported coronary heart disease; dbp, logarithm of diastolic blood pressure; dm, self-reported diabetes; dmd, self-reported diabetes and following a diet; dmm, self-reported diabetes and taking medication; edu, education; fpg, logarithm of fasting plasma glucose; fr, frequency of fruit consumption; hba1c, logarithm of Haemoglobin A1c in %; hbp, self-reported high blood pressure; hbpd, self-reported high blood pressure and following a diet; hbpm, self-reported high blood pressure and taking medication; hcl, self-reported hypercholesterolemia (high cholesterol levels); hcld, self-reported hypercholesterolemia and following a diet; hclm, self-reported hypercholesterolemia and taking medication; hdl logarithm of High Density Lipoprotein cholesterol in mg/dl; in, income; ltpa, leisure-time physical activity; mi, self-reported myocardial infarction; nat, nationality; nonhdl, logarithm of non-High Density Lipoprotein cholesterol in mg/dl; oshd, self-reported other serious heart disease; sbp, logarithm of systolic blood pressure; smk, smoking status; snck, frequency of snacking; ssbs, frequency of sugar-sweetened beverage consumption; strk, self-reported stroke; tc, logarithm of total cholesterol in mg/dl; tg, logarithm of triglycerides in mg/dl; veg, frequency of vegetable consumption; waist, logarithm of waist circumference in cm.*

**Table 3** Missing (%) values in selected variables from the BHIS2018 (adults), and predictors selected for imputation using the Predictor Matrix function of the R package *mice*

| **Variable** | **Variable type** | **Missing (%)** | **Additional variables, other than age, sex, and province, to inform imputation** |
| --- | --- | --- | --- |
| Nationality | Categorical (3 levels) | 4 (0.04%) | - |
| Household education level | Categorical (4 levels) | 179 (1.9%) | in, fr, veg, alc, ltpa, height, bmi, hbp, hbpm, dm, dmm,hcl, hclm |
| income quintile | Categorical (5 levels) | 1,527 (16%) | edu, veg, snck, alc, ltpa, height, hbp, hbpm, dm, dmm, hcl,hclm |
| Frequency of fruit consumption | Categorical (5 levels) | 7 (0.07%) | edu, veg, ssbs, smk, alc, ltpa |
| Frequency of vegetable consumption | Categorical (5 levels) | 5 (0.05%) | edu, smk, ltpa |
| Frequency of sugar-sweetened beverage consumption | Categorical (5 levels) | 6 (0.06%) | smk, height, hbpm, dm, dmm |
| Frequency of snacking | Categorical (5 levels) | 8 (0.08%) | in, ssbs, dm, dmd, dmm |
| Smoking status | Categorical (3 levels) | 1,823 (19%) | nat, edu, fr, veg, ssbs, height |
| Distribution of alcohol consumption | Categorical (5 levels) | 1,954 (21%) | nat, edu, in, fr, height, weight |
| Leisure-time physical activity | Categorical (3 levels) | 2,183 (23%) | nat, edu, in, fr, veg, height, bmi, hbp, hbpm, dm, dmm |
| Self-reported height | Continuous | 49 (0.52%) | edu, in, ssbs, smk, alc, ltpa, weight |
| Self-reported weight | Continuous | 119 (1.3%) | alc, height, hbp, hbpm, dm, dmm |
| Self-reported BMI | Continuous | 144 (1.5%) | Calculated from self-reported weight and height |
| Self-reported high blood pressure | Categorical (2 levels) | 6 (0.06%) | edu, in, ltpa, weight, bmi, dm, dmd, dmm, hcl, hcld, hclm, oshd |
| Self-reported high blood pressure and following a diet | Categorical (2 levels) | 6 (0.06%) | bmi, dm, dmd, dmm, hcl, hcld, hclm, oshd |
| Self-reported high blood pressure and taking medication | Categorical (2 levels) | 6 (0.06%) | edu, in, ssbs, ltpa, weight, bmi, dm, dmd, dmm, hcl, hcld, hclm, oshd |
| Self-reported diabetes | Categorical (2 levels) | 3 (0.03%) | edu, in, ssbs, snck, ltpa, weight, bmi, hbp, hbpd, hbpm, hcl, hcld, hclm |
| Self-reported diabetes and following a diet | Categorical (2 levels) | 3 (0.03%) | snck, bmi, hbp, hbpd, hbpm, hcl, hcld, hclm, chd |
| Self-reported diabetes and taking medication | Categorical (2 levels) | 3 (0.03%) | edu, in, ssbs, snck, ltpa, weight, bmi, hbp, hbpd, hbpm, hcl, hcld, hclm |
| Self-reported high cholesterol level | Categorical (2 levels) | 33 (0.35%) | edu, in, bmi, hbp, hbpd, hbpm, dm, dmd, dmm, chd, oshd |
| Self-reported high cholesterol levels and following a diet | Categorical (2 levels) | 33 (0.35%) | hbp, hbpd, hbpm, dm, dmd, dmm |
| Self-reported high cholesterol levels and taking medication | Categorical (2 levels) | 33 (0.35%) | edu, in, bmi, hbp, hbpd, hbpm, dm, dmd, dmm, MI, chd, oshd |
| Self-reported coronary heart disease | Categorical (2 levels) | 2 (0.02%) | dmd, hcl, hclm, mi, oshd, strk |
| Self-reported other serious heart disease | Categorical (2 levels) | 1 (0.01%) | hbp, hbpd, hbpm, hcl, hclm, mi, chd, strk |
| Self-reported stroke | Categorical (2 levels) | 1 (0.01%) | chd, oshd |
| Self-reported cancer | Categorical (2 levels) | 1 (0.01%) | - |

*Abbreviations: alc, distribution of alcohol consumption; chd, self-reported coronary heart disease; dbp, logarithm of diastolic blood pressure; dm, self-reported diabetes; dmd, self-reported diabetes and following a diet; dmm, self-reported diabetes and taking medication; edu, education; fpg, logarithm of fasting plasma glucose; fr, frequency of fruit consumption; hba1c, logarithm of Haemoglobin A1c in %; hbp, self-reported high blood pressure; hbpd, self-reported high blood pressure and following a diet; hbpm, self-reported high blood pressure and taking medication; hcl, self-reported hypercholesterolemia (high cholesterol levels); hcld, self-reported hypercholesterolemia and following a diet; hclm, self-reported hypercholesterolemia and taking medication; hdl logarithm of High Density Lipoprotein cholesterol in mg/dl; in, income; ltpa, leisure-time physical activity; mi, self-reported myocardial infarction; nat, nationality; nonhdl, logarithm of non-High Density Lipoprotein cholesterol in mg/dl; oshd, self-reported other serious heart disease; sbp, logarithm of systolic blood pressure; smk, smoking status; snck, frequency of snacking; ssbs, frequency of sugar-sweetened beverage consumption; strk, self-reported stroke; tc, logarithm of total cholesterol in mg/dl; tg, logarithm of triglycerides in mg/dl; veg, frequency of vegetable consumption; waist, logarithm of waist circumference in cm.*

**Table 4** Missing (%) values in selected variables from the BHIS2018 (children), and predictors selected for imputation using the Predictor Matrix function of the R package *mice*

| **Variable** | **Variable type** | **Missing (%)** | **Additional variables, other than age, sex, and province, to inform imputation** |
| --- | --- | --- | --- |
| Nationality | Categorical (3 levels) | 1 (0.05%) | edu, in, ltpa |
| Household education level | Categorical (4 levels) | 30 (1.4%) | nat, in, fr, veg, ssbs |
| income quintile | Categorical (5 levels) | 316(15%) | nat, edu, veg, ssb, bmi |
| Frequency of fruit consumption | Categorical (5 levels) | 2 (0.09%) | edu, veg, ssbs |
| Frequency of vegetable consumption | Categorical (5 levels) | 2 (0.09%) | edu, in, fr, ssbs, ltpa |
| Frequency of sugar-sweetened beverage consumption | Categorical (5 levels) | 1 (0.05%) | edu, in, fr, ltpa |
| Frequency of snacking | Categorical (5 levels) | 1 (0.05%) | ltpa |
| Smoking status | Categorical (3 levels) | 101 (4.7%)^1^ | fr, height, weight |
| Distribution of alcohol consumption | Categorical (5 levels) | 99 (4.6%)^1^ | fr, height, weight |
| Leisure-time physical activity | Categorical (3 levels) | 1,888 (87%)^1^ | nat, edu, in, fr, veg, ssbs, smk, alc, bmi |
| Self-reported height | Continuous | 73 (3.4%) | fr, ssbs, snck, smk, alc, weight |
| Self-reported weight | Continuous | 68 (3.1%) | fr, ssbs, snck, smk, alc, height |
| Self-reported BMI | Continuous | 108 (5.0%) | Calculated from self-reported weight and height |

*Abbreviations: alc, distribution of alcohol consumption; fr, frequency of fruit consumption; in, income; ltpa, leisure-time physical activity; nat, nationality; smk, smoking status; snck, frequency of snacking; ssbs, frequency of sugar-sweetened beverage consumption; veg, frequency of vegetable consumption. ^1^ Information on smoking status, alcohol consumption and leisure-time physical activity was collected from the age of 15 years onwards, and therefore smoking status was set to “never smoked” and distribution of alcohol consumption to “abstainers” for the ages 0 to 14 years of age, while leisure-time physical activity was imputed for those ages, except for the age of 0 where it was set to sedentary.*

# Model structure

The T2D-M is implemented as a discrete-event state transition model with two events, i.e., incident T2D or death, updated annually, while assuming hierarchy of events. In each annual cycle, after updating age and the prevalence of risk factors, each synthetic individual can transition from non-T2D to T2D and/or to death, or from T2D to death. Transition probabilities for T2D are estimated based on individual socio-demographic characteristics and prevalent risk factors updated yearly via strata-specific transition probabilities. Transition probabilities for death are estimated based on strata-specific transition probabilities adjusted for T2D status. Throughout the simulation period, and in each annual cycle, the level of risk factors, T2D and survival status are tracked for each individual.

## Age update

As simulation progresses in annual cycles, the age of each synthetic individual in the cohort increases by one year in each model cycle, but all other socio-demographics characteristics (including sex, province of residence, educational level and income level) remain the same over the simulation period. Therefore, the current implementation of the T2D-M simulates the result of aging, disregarding any potential influence of socio-demographical movements due to the lack of details to inform the model inputs ((see heading 8, list of assumptions).

## Risk factors update

The relevant risk factors for predicting T2D status (BMI, waist circumference, use of BP medication and history of high blood glucose levels) are considered modifiable risk factors, and are assumed to evolve over time. To account for the possibility of an individual moving from one risk factor level (state) to another in a year, the transition probabilities of each risk factor were estimated using the methodology developed by van de Kassteele et al. (24) that allows the estimation of net-transition probabilities for categorical variables from survey data. Relevant risk factors in the T2D-M were recoded categorically matching the risk factors levels (states) of FINDRISC (13). For children (aged 0-17 years) the current implementation of the model only considers updating BMI yearly with risk factors levels (states) based on Z-scores (21, 22) to categorise overweight and obesity. However, when becoming the age of 18, an update of the waist circumference was based on a sex-BMI-specific transition probabilities, while use of BP medication and history of high blood glucose levels were assumed to be not present. The annual update of a given categorical risk factor described, for example, according to three states (normal, medium, and high-risk factor levels) is carried out in two steps using the following notation:

Let k represent the total number of states (risk factor levels), *a* denote age, *i* state at age a-1; *j* state at age a; *τ_ij_(a)* the transition (in prevalence) from state *i* at age *a-1* into state *j* at age *a*; τ’_ij_(a) the net transition from state *i* at age *a-1* into state *j* at age *a*; *π_j_(a)* and *π_i_(a-1)* are how likely an individual is in state *j* at age *a*, or state *i* at age *a-1* respectively; *p_ij_(a)* is the transition probability from state *i* at age *a-1* to state *j* at age *a*; *p'_ij_(a)* is the net transition probability from state *i* at age *a-1* to state *j* at age *a*.

In step 1, the prevalence (proportion) $\hat{\pi}_{j}(a)$ of individuals in the different states (normal, medium and high-risk) by strata is estimated and smoothened using a binomial or multinomial P-spline, dependent on the number of states of the risk factor (i.e. dichotomous or multiple states), using the R package “*mgcv”* (5). In step 2, the net-transitions between states, ${\tau^{'}}_{ij}(a)$, are estimated through a transportation optimisation algorithm in the R package *“lpSolve*” (4). Here, the prevalence at the current age provides demand, $\hat{\pi}_{j}(a)$, that is supplied by the prevalence in the previous age $\hat{\pi}_{i}(a-1)$ at a cost c_ij_. This variable indicates the cost for an individual to move from one state to another in the next year. A symmetric k by k Toeplitz matrix (where k is the total risk factor level) is used as a cost matrix. In this matrix, the cost of remaining in the same state is zero, the cost of moving to one state up or down is one unit and cost of moving two states up or down is three units. With this setting, it is more likely (e.g. inexpensive) for an individual to remain in his/her current risk factor state than moving one state up or down, and very unlikely (e.g. expensive) to move two states up or down (that is from normal to high risk, or vice versa) in only one annual cycle. In addition, due to the small probabilities for individuals to change BMI or waist circumference categories (from normal to medium to high risk, and vice versa) in one year, a transition if at least 2.5% of the indivudals was forced to make this change in risk factors more notable (see heading 8, list of assumptions). For the use of BP medication and history of high blood glucose, no transition to “risk factors not present” was allowed once “on medication” or “having history”, respectively, occurred. These extra restrictions for estimating the transition probabilities were added as an additional constraint to the respective transportation optimisation algorithm. The net transitions are then estimated by minimising the objective function *J* using simplex method in linear programming, as follows:

$$J=\sum_{i =1}^{k} \sum_{j=1}^{k} A=c_{ij} {\tau^{'}}_{ij}\left( a \right)$$

subject to:

$\sum_{j=1}^{k} {\tau^{'}}_{ij}(a)= \hat{\pi}_{i} (a-1)$, $\sum_{i=1}^{k} {\tau^{'}}_{ij}(a)= \hat{\pi}_{j} \left( a \right),$ and ${\tau^{'}}_{ij}(a)\geq0$

By using the simplex method, net transitions ${\tau^{'}}_{ij}(a)$ are obtained where for i ≠j

$${\tau^{'}}_{ij}(a)=\{\tau_{ij}\left( a \right)- \tau_{ji}(a), \tau_{ij}> \tau_{ji} 0, otherwise$$

for

$i=j$, ${\tau^{'}}_{ij}\left( a \right)=\pi_{i}\left( a-1 \right)- \sum_{j\neq i}^{k} {\tau^{'}}_{ij}(a)$

The net transition probabilities at age *a* are then estimated by dividing the net transitions at age *a* by the smoothened prevalence at age *a-1* that is

${p^{'}}_{ij}\left( a \right)= \frac{{\tau^{'}}_{ij}(a)}{\hat{\pi}_{i} (a-1)}$ .

The net transition probabilities for each risk factor are estimated with corresponding 95% confidence intervals, by generating 1,000 parameter values from a multivariate normal distribution with mean and covariance equals to the coefficients and variance covariance matrix of the multinomial P-spline model fitted on the risk factor categories in step 1 respectively. Subsequently, with these 1,000 parameter values, 1,000 transition probabilities are generated, and the lower and upper confidence interval calculated by taking the 2.5% and 97.5% quantiles, respectively (**Figures 2-5**). For the current implementation of the T2D-M, the mean of the 1,000 transition probabilities generated was taken to simulate the update of the risk factor states.

The net transition probabilities were estimated using the BHIS-2018 and BHES-2018, and incorporated time-trends as estimated from the repeated cross-sectional survey data [BHIS 1997, 2001, 2004, 2008, 2013 and 2018]. Hence transition probabilities for risk factors were inflated for time following the approach of Fleurence et al. (25). After transforming the risk factor transition probabilities to rates, rates are multiplied by the strata-specific relative risks of time calculated as the exponentiated beta that corresponds to the simulation year of interest (**Table 5** and **Table 6**), and then transformed back to a one-year risk factor transition probabilities adjusted for time. In each simulation year and for each risk factor, the risk factor state update is based on drawing from a bi- or multinomial distribution using the strata-specific net-transition probabilities adjusted for the simulation year of interest. In R, this is modelled using *rbinom* or *rmultinom*.

**Figure 2** Stratified net transition probabilities and corresponding 95% confidence intervals for BMI groups: normal (BMI <25 kg/m²), overweight (BMI 25-30 kg/m²), or obese (BMI >30 kg/m²)


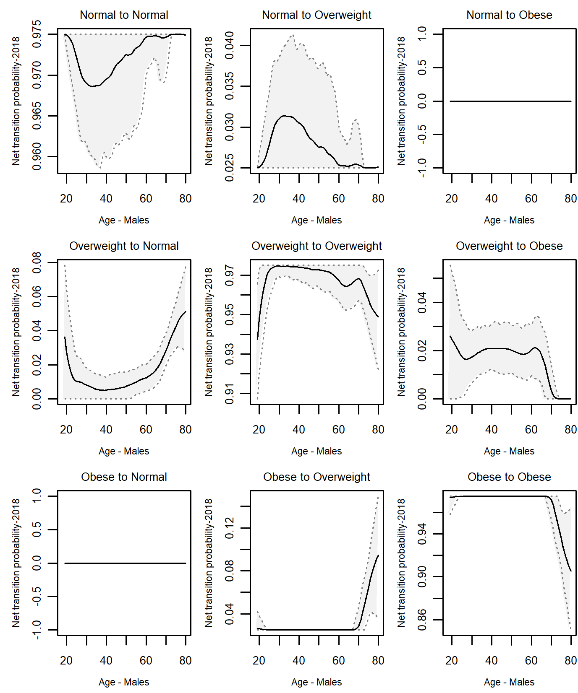

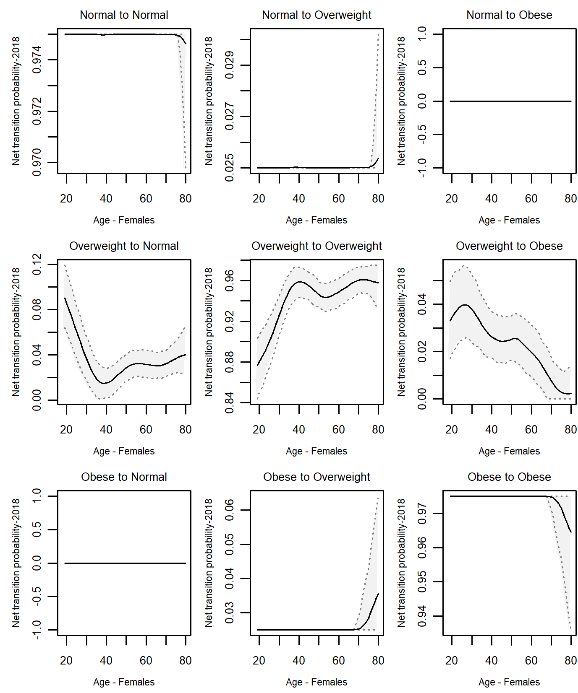


**Figure 3** Stratified net transition probabilities and corresponding 95% confidence intervals for waist circumference groups: normal (men: <94 cm; women: <80 cm), medium risk (men: 94-102 cm; women: 80-88 cm), or high risk (men: ≥102 cm; women: ≥88 cm)


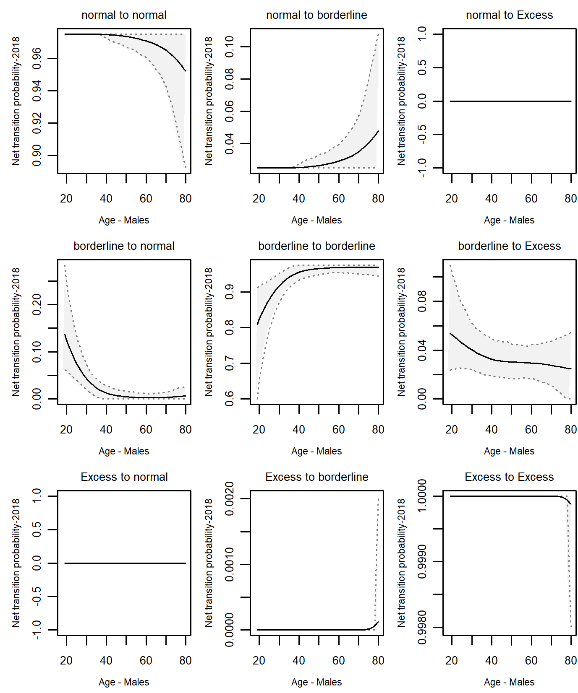

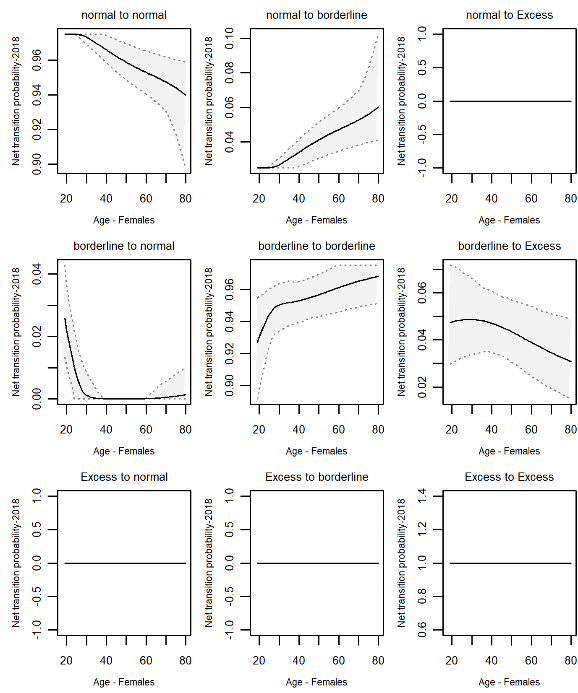


**Figure 4** Stratified net transition probabilities and corresponding 95% confidence intervals for blood pressure based on reports of current medication


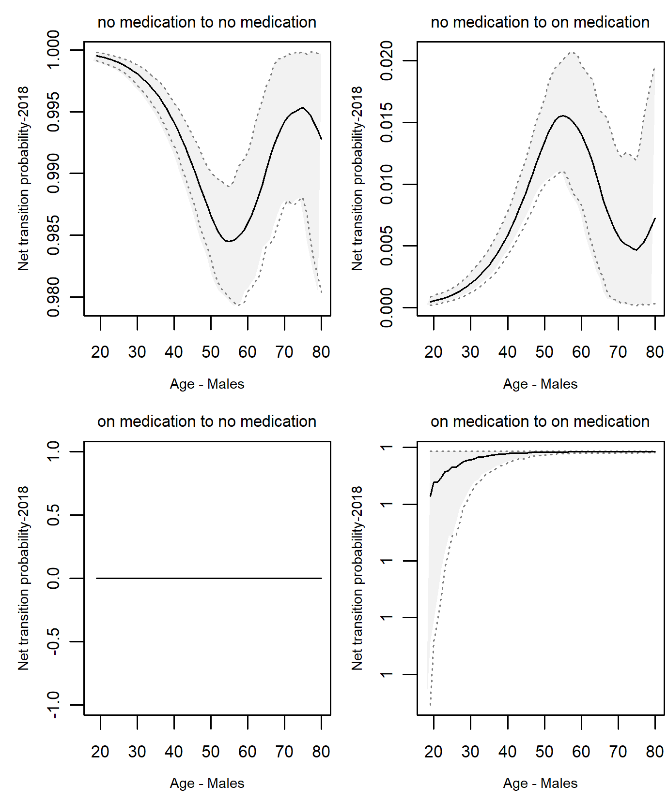

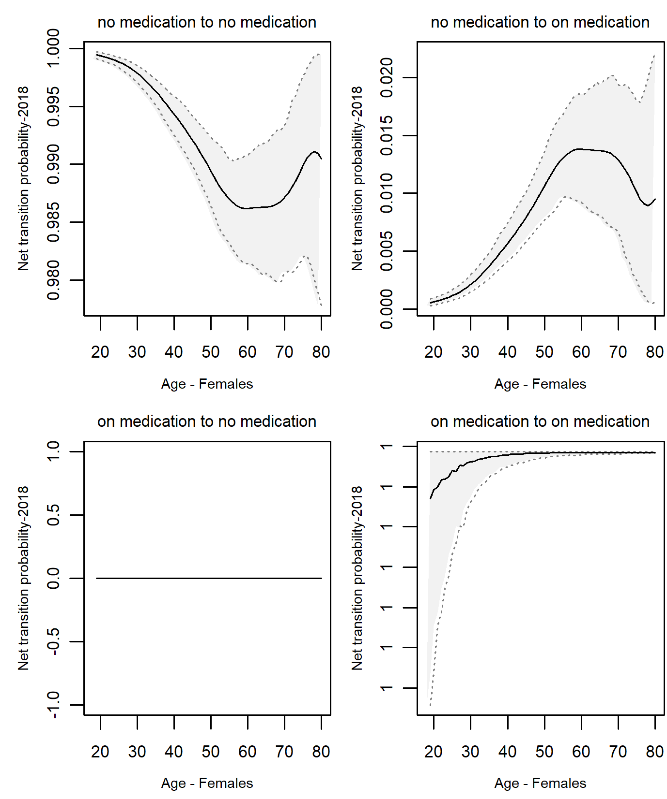


**Figure 5** Stratified net transition probabilities and corresponding 95% confidence intervals for blood glucose based on reports of history of blood glucose


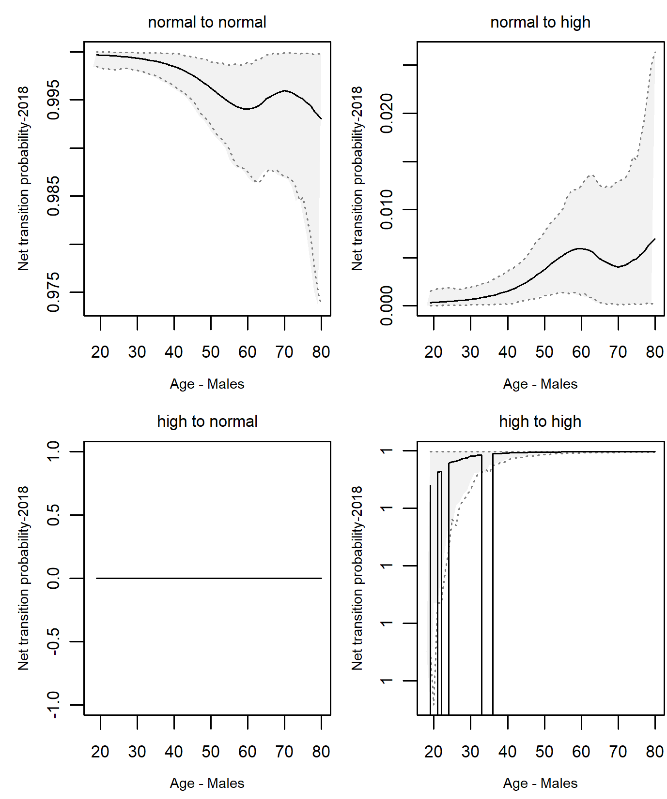

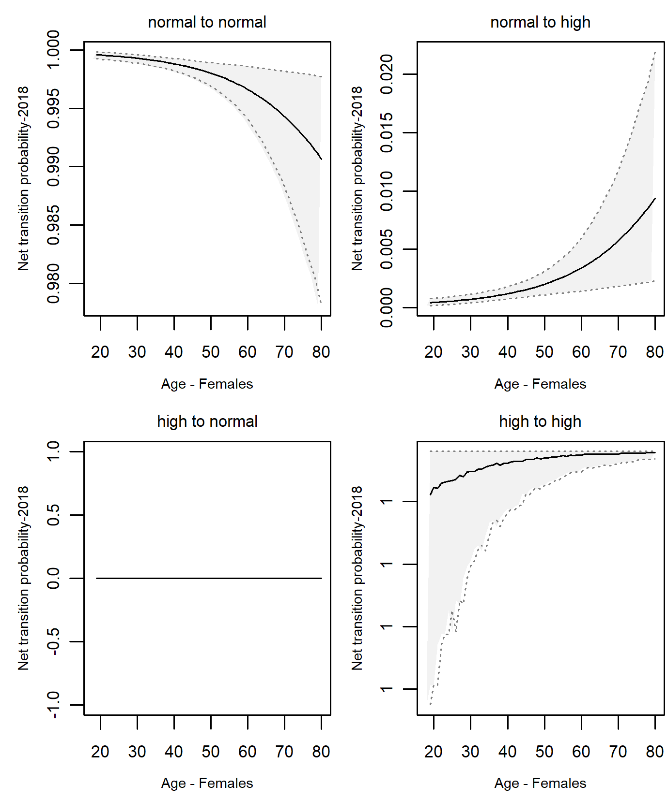


**Table 5** Strata-specific beta coefficients for time, as estimated from an age-adjusted survey-weighted logistic model using all available BHIS waves to further inflate the transition probabilities for adult population, aged 18 years and older, accounting for time trends*^1,2^*

| **Sex** | **Region** | **Education** | **BMI** | | | **Use BP medication** | | **History of blood glucose** | |
| --- | --- | --- | --- | --- | --- | --- | --- | --- | --- |
|  |  |  | **Normal** | **Overweight** | **Obesity** | **Yes** | **No** | **Yes** | **No** |
| Men | Flanders | Low | -0.081 | -0.029 | 0.178 | 0.195 | -0.195 | 0.113 | -0.113 |
|  |  | Intermediate | -0.050 | -0.011 | 0.132 | 0.230 | -0.230 | 0.207 | -0.207 |
|  |  | High | -0.074 | 0.044 | 0.103 | 0.197 | -0.197 | 0.319 | -0.319 |
|  | Brussels | Low | -0.079 | 0.069 | 0.022 | 0.028 | -0.028 | 0.222 | -0.222 |
|  |  | Intermediate | -0.157 | 0.083 | 0.160 | 0.127 | -0.127 | 0.271 | -0.271 |
|  |  | High | -0.072 | 0.022 | 0.145 | 0.211 | -0.211 | 0.140 | -0.140 |
|  | Wallonia | Low | -0.052 | -0.006 | 0.087 | 0.122 | -0.122 | 0.083 | -0.083 |
|  |  | Intermediate | -0.032 | 0.009 | 0.038 | 0.109 | -0.109 | 0.142 | -0.142 |
|  |  | High | -0.044 | -0.003 | 0.101 | 0.227 | -0.227 | 0.063 | -0.063 |
| Women | Flanders | Low | -0.086 | 0.042 | 0.071 | 0.065 | -0.065 | 0.180 | -0.180 |
|  |  | Intermediate | -0.091 | 0.053 | 0.096 | 0.209 | -0.209 | 0.146 | -0.146 |
|  |  | High | -0.095 | 0.062 | 0.131 | 0.213 | -0.213 | 0.131 | -0.131 |
|  | Brussels | Low | -0.137 | 0.077 | 0.100 | 0.125 | -0.125 | 0.209 | -0.209 |
|  |  | Intermediate | -0.191 | 0.138 | 0.160 | 0.187 | -0.187 | 0.215 | -0.215 |
|  |  | High | -0.065 | 0.051 | 0.067 | 0.111 | -0.111 | 0.202 | -0.202 |
|  | Wallonia | Low | -0.063 | 0.006 | 0.081 | 0.108 | -0.108 | 0.013 | -0.013 |
|  |  | Intermediate | -0.077 | 0.071 | 0.033 | 0.059 | -0.059 | 0.071 | -0.071 |
|  |  | High | -0.083 | 0.015 | 0.172 | 0.115 | -0.115 | 0.252 | -0.252 |

*^1^ Beta coefficients for time represents the expected change in log odds of having the outcome for a 5-year time change (i.e. time-interval between the BHIS survey cycles). ^2^Strata-specific time-trends of BMI were assumed to be also applicable for inflating transition probabilities of waist circumferences for time, and those of diabetes for history of high blood glucose.*

**Table 6** Strata-specific beta coefficients for time, as estimated from an age-adjusted survey-weighted logistic model using all available BHIS data, to further inflate the transition probabilities for children, accounting for time trends*^1,2^*

| **Sex** | **Region** | **Education** | **BMI normal** | **Overweight** | **Obesity** |
| --- | --- | --- | --- | --- | --- |
| Men | Flanders | Low | -0.098 | -0.002 | 0.165 |
|  |  | Intermediate | -0.037 | 0.029 | 0.037 |
|  |  | High | -0.058 | 0.101 | -0.025 |
|  | Brussels | Low | -0.128 | 0.132 | 0.069 |
|  |  | Intermediate | 0.072 | -0.210 | 0.149 |
|  |  | High | -0.041 | 0.076 | -0.009 |
|  | Wallonia | Low | -0.060 | 0.028 | 0.068 |
|  |  | Intermediate | -0.055 | -0.028 | 0.174 |
|  |  | High | -0.042 | 0.010 | 0.104 |
| Women | Flanders | Low | -0.082 | -0.010 | 0.156 |
|  |  | Intermediate | -0.184 | 0.141 | 0.223 |
|  |  | High | 0.019 | -0.031 | 0.013 |
|  | Brussels | Low | -0.048 | 0.020 | 0.056 |
|  |  | Intermediate | -0.088 | 0.076 | 0.070 |
|  |  | High | 0.033 | -0.026 | -0.032 |
|  | Wallonia | Low | -0.149 | 0.205 | 0.039 |
|  |  | Intermediate | -0.181 | 0.170 | 0.133 |
|  |  | High | 0.035 | -0.109 | 0.243 |
| Sex | BMI group |  | BMI |  |  |
|  |  |  | Normal | Overweight | Obesity |
| Men | Normal |  | 0.025 | -0.025 | -0.025 |
|  | Overweight |  | -0.025 | 0.025 | -0.025 |
|  | Obesity |  | -0.009 | -0.009 | 0.009 |
| Women | Normal |  | 0.022 | -0.022 | -0.022 |
|  | Overweight |  | -0.028 | 0.028 | -0.028 |
|  | Obesity |  | -0.003 | -0.003 | 0.003 |

*^1^ Beta coefficients for time represents the expected change in log odds of having the outcome for a 5-year time change (i.e. time-interval between the BHIS survey cycles).  ^2^ Strata-specific time-trends of BMI were assumed to be also applicable for adjusting transition probabilities of waist circumferences for time, provided that they were estimated for the same strata in which the transition probabilities were calculated.*

## T2D status update

In the T2D-M, the probability of developing diabetes is calculated for each individual, aged 18 years and older, using the concise version of the FINDRISC (13) equation, a widely-used validated score to predict the 10-year T2D incidence in European populations, including Belgium (18). The point estimates of the coefficients of the respective risk factors fitted in a logistic regression to estimate an individual’s 10-year probability:

$$p\left( {diabetes}_{i} \right)= \frac{e^{( \beta_{0}+ \beta_{1}x_{i1}+ \beta_{2}x_{i2}+\ldots+ \beta_{p}x_{ip})}}{1+ e^{( \beta_{0}+ \beta_{1}x_{i1}+ \beta_{2}x_{i2}+\ldots+ \beta_{p}x_{ip})}}$$

where $\beta_{0}$ $\beta_{1}$, $\beta_{2}$, ..., $\beta_{p}$ are the regression coefficients for the $p$ risk factors represented by $x_{1}$ $x_{2}$, ..., $x_{p}$and $i=1,2,3,\ldots, n$ refers to the ${i^{th}}^{'}s$ individual.

This 10-year probability is transformed to a one-year probability using the method described by Fleurence et al. (25). Under the assumption of no competing risks (see heading 8, list of assumptions), the 10-year probability is transformed to annual rates using the formula $r= -loglog \left( 1-p\left( {diabetes}_{i} \right) \right)$, where $p\left( {diabetes}_{i} \right)$ is the 10-year probability and r the annual rate, and then the annual rates transformed to annual probability using the formula $p\left( {diabetes}_{i1yr} \right)=1- e^{-r*t}$ where $p\left( {diabetes}_{i1yr} \right)$ is the annual probability and t = 1. Subsequently, the incidence of T2D is based on drawing from a binomial distribution using the one-year probability. Once an individual has diabetes, either at baseline or developed over the simulation period, they will have diabetes over the whole simulation period or until they die.

## Survival status update

All individuals in the model are exposed to the risk of dying in each model cycle. Data on diabetes-specific and all-cause mortality in Belgium was obtained from SPMA (11) for the years 1987-2018, with total number of death and population stratified by five-years age groups, sex and province. The mortality rates for each population stratum were calculated by dividing total deaths by total population (all-causes) or total non-diabetes death (calculated as all cause minus diabetes-specific cause) by total population (other cause). In the first step, we estimate the age-specific (0-100 years old) mortality rates from the five year age group mortality rates by using the monotonic smoothing spline method implemented in the R package *demography* (6). In a second step, forecasting the age-specific mortality rates (2019-2030) is performed using the smoothened age-specific mortality rates as input in a functional demographic model (26) implemented in the R package *demography* (6). In the third step, we estimate the age-specific probability of death stratified by province *p* and sex *s* by using the resulting age-specific, sex, province mortality rates (${smoothMR}_{asp}$) as input in the formula (25):

$$p\left( {death}_{asp} \right)=1- e^{-{smoothMR}_{asp} *1}$$

Accounting for the increased risk of all-cause mortality of people with T2D, the age-sex-specific relative risk of death from the DECODE study (14) was used to adjust the estimated probability of death following the approach of Fleurence et al. (25) (see heading 8, list of assumptions). In brief, after transforming the probability of death to a death rate, this rate is multiplied by the 10-year age-group, sex-specific relative risk of death, and then transformed back to a one-year probability adjusted for T2D status. In this way, an individual with T2D has a higher probability of all-cause death compared to a similar individual with same age, sex and province without T2D. Similar to the update of the T2D status, the probability of death is used to determine the update survival status by drawing from a binomial distribution. The probability of death for ages ≥ 100 was set to one, so that no one in the model lives beyond the age of 100.

# Model output

The main model output is an annual forecast of the burden of T2D in terms of incidence and prevalence during the whole simulation period. The burden of T2D was estimated calculating disability-adjusted life year (DALYS), a time-based statistic combining the years of life lost (YLL) due to premature mortality and the years of life lost due to time lived in states of less than full health, or years of healthy life lost due to disability (YLDs) and so aggregating morbidity and mortality in a single metric.

$$DALY=YLL+YLD$$

$YLD= \sum_{i=1}^{a} \sum_{j=1}^{s} \sum_{k}^{c} D_{i,j}*P_{i, j, k}*{DW}_{k}$ $YLL= \sum_{i=1}^{a} \sum_{j=1}^{s} \sum_{l=1}^{r} M_{i,j,l}*{RLE}_{i, j, l}$

Where *i*=1…a represent specified age groups; *j*=1…s specified sex; *k*=1…c specified T2D complications; *D_i, j_* is the age-sex specific number of individuals with T2D; *P _i, j, k,_* the age-sex specific prevalence of the T2D complication *k*; *DW_k_* the associated disability weight (DW) for T2D complication *k*; l=1…r specified regions; *M_i, j, l_* is the age-sex-and region-specific number of deaths due to T2D; and *RLE_i, j, l_*, the age-sex-region-specific residual life expectancy.

While the age-sex specific number of individuals with T2D is a direct output of the T2D-M, the number of deaths due to T2D is indirectly calculated via the total number of deaths multiplied by the proportion mortality rate for T2D taken from SPMA for the year 2019, specific for the relevant age, sex and region groups.

For the calculation of the YLD, in alignment with the Belgian Burden of Disease protocol (27), the relevant health states for T2D were: uncomplicated diabetes, diabetic neuropathy, diabetic neuropathy with diabetic foot, diabetic neuropathy with amputation (including with and without treatment), vision impairment (including moderate and severe) and blindness, with associated DW taken from the Global Burden of Disease study (GBD). The GBD study provides a comprehensive overview of mortality and disability across countries, time, age and sex for a list of mutually exclusive and collectively diseases and injuries. GBD produces estimates for diseases and injuries in terms of all cause mortality, deaths by cause, years of life lost due to premature mortality (YLLs), YLDs, and disability-adjuted life years (DALYs) (28). The proportion of T2D cases in the respective health states by age and sex groups were derived from Belgian data from the Initiative for Quality Improvement and Epidemiology in Diabetes (IQED) study. Briefly, in this study, data were collected retrospectively from March 1, 2020 until February 28, 2021, among specialists in diabetic centers (N=103) on both people with type I diabetes (T1D) and T2D aged 16 years or more who injected insulin at least three times a day, hereby covering virtually all T1D patients, but only the T2D on insulin treatment. Each center provided a 10% patient sample with a minimum sample of 50 patients, conforming a study population of 7,808 diabetic individuals, with survey weights applied to ensure representativeness of the diabetic population visiting the centers (29). For the calculation of YLL, Belgian life tables based on population observations from the National Register (births, deaths and population numbers), as published by STATBEL (8), were used to retrieve the residual life expectancy by age, sex and region. Estimations of 95% CI were obtained taking the lower and upper bounds for the age-sex specific number of estimated individuals with T2D (*D_i, j_*), the age-sex specific prevalence of the T2D-specific complication *k* (*P _i, j, k,_*), the associated DW for T2D-specific complication *k* (*DW_k_*), and the age-sex-and region-specific number of deaths due to T2D (*M_i, j, l_*).

In addition, T2D risk factors i.e. BMI (overweight and obesity), elevated waist circumference, elevated BP and high blood sugar (measured as current medication or previous history, respectively), and their annual, and overall changes during the simulation, are also outputs of the model. Because the predictive risk function that determine the model risk for T2D (FINDRISC) was developed primarily for adults aged 35+ years, the model outputs for incident and prevalent T2D are presented for this age group onwards. Notably, because very few events occur before this age (1.4% among those aged 18-34 years vs 5.1% and 13.8% in adults aged 35-64 years and ≥65 years, respectively), excluding the young adults would not influence findings (see heading 8, list of assumptions). For children and the young adults (aged 35 or less), the main model output is an estimate of the burden of overweight and obesity in terms of incidence and prevalence, annually, and over the simulation period.

# Model uncertainty

The T2D-M integrates four types of uncertainty in the uncertainty intervals of the output’s estimates. i.e., individual heterogeneity and structural, stochastic, parameter uncertainty (30). However, due to a lack of information and for computational efficiency, it is not feasible to fully account for four types of uncertainty throughout the whole microsimulation process.

## Individual heterogeneity

Individual heterogeneity refers to the between-individual variability attributable to differences in individual characteristics, and hence regarded as the not completely random between-individual variation that is accounted for by allowing probabilities to be conditional on individual characteristics (i.e. age, sex, province, exposure to risk factors, or disease diagnosis). In the T2D-M, individual heterogeneity is accounted for by using strata-specific transition probabilities for updating risk factor states, risk-factor-specific transition probabilities, as integrated in a risk prediction equation, for updating T2D status, and age-sex-province-T2D-specific probabilities for updating survival status.

## Structural uncertainty

Structural uncertainty, referring to the type of uncertainty related to the assumptions inherent in the model, include all the simplifications and scientific judgements made when building the framework and interpreting the outputs. For example, the decision about which risk factors/events to include and the procedure for updating them due to aging, and time are made in a certain order. In the T2D-M, a hierarchy of events is assumed with annual updates only due to aging, and since disregarding the competing events between T2D and death as well as any time effect in risk factor trends was considered to have a minimal impact on the model results, its penitential impact was not evaluated (see heading 8, list of assumptions).

## Stochastic uncertainty

Stochastic uncertainty refers to inherent variability in model outcome between identical individuals, and is due to the random sampling of event manifestations for every simulation annual run. In T2D-M, a random draw from a Bernoulli/multinomial probability distribution with a particular probability is used for deciding whether the event will manifest or not in that particular individual in that particular year. This type of uncertainty is included throughout the whole simulation from updating risk factor states to incident T2D and survival status, and this for all individuals in all the model cycles.

## Parameter uncertainty

Parameter uncertainty refers to the uncertainty of input parameters introduced by sampling errors, and is accounted for by running multiple iterations and using for each iteration a different set of input parameters, as taken by sampling from respective distributions of input parameters. This type of uncertainty is the only that can be reduced from future improved parameters. In individual-level model, probabilistic sensitivity analyses (PSA), assessing the magnitude of parameter uncertainty, are implemented as a double loop: the number of iterations (outer loop) and the number of individuals per iteration (inner loop).

In the T2D-M, the uncertainty intervals for the incidence and prevalence of T2D were estimated from 100 iterations of the model, repeatedly sampling from the input parameter distributions of the risk prediction equation for developing T2D, i.e. the FINDRISC, and in a different sample of 1% of the synthetic individuals. The current implementation of the T2D-M only considered parameter uncertainty for the risk prediction equation for developing T2D. In particular, uncertainty in the FINDRISC coefficients was incorporated assuming a uniform distribution (β ~ unif(a, b)) with parameters equal to the estimated regression coefficients and their confidence limits: a, the minimum value taken from the lower confidence limit, and b, the maximum value taken from the upper confidence limit and minimum values were bounded above 0 when mean above 0. In contrast, parameter uncertainty for the input parameters of transition probabilities for risk factor states and survival states was considered as relatively small. This is because for the risk factor transition probabilities the mean of 1,000 replicates was used and their 95% CI were narrow, and for mortality probabilities based on smoothing and forecasting with underlying observed mortality data covering more than 30 years.

# Model validation

## Validation of the synthetic population

The similarity of the synthetic population generated and the corresponding (real) Belgian population of 2018 was evaluated as internal validation of the model. Intraclass coefficients (ICC) computed for the age-sex-province group on the prevalence of risk factors and T2D as retrieved from the synthetic population generated, and those from the latest BHIS and BELHES of 2018, were used as a measure of agreement. The ICC results (**Table 7**) support the agreement between the T2D-M synthetic population and the nationally representative survey data.

**Table 7** Intra-class correlation coefficients and 95% confidence interval of the comparison between age-sex-province group estimates as retrieved from the synthetic population or the BHIS-2018 and BHES-2018 surveys

| **Variable** | **Variable collected in** | **Population group** | **ICC** | **(95%CI)** |
| --- | --- | --- | --- | --- |
| Nationality | BHIS2018 | All | 0.997 | (0.997, 0.997) |
| Education level | BHIS2018 | All | 0.974 | (0.970, 0.978) |
| Income quintiles | BHIS2018 | All | 0.888 | (0.876, 0.899) |
| Self-reported alcohol consumption | BHIS2018 | All | 0.903 | (0.891, 0.914) |
| Self-reported smoking status | BHIS2018 | All | 0.905 | (0.891, 0.917) |
| Self-reported leisure-time physical activity | BHIS2018 | All | 0.808 | (0.779, 0.835) |
| Self-reported frequency of fruit | BHIS2018 | All | 0.987 | (0.986, 0.988) |
| Self-reported frequency of vegetables | BHIS2018 | All | 0.993 | (0.992, 0.993) |
| Self-reported frequency of sugar-sweetened beverages | BHIS2018 | All | 0.963 | (0.959, 0.967) |
| Self-reported frequency of snacking | BHIS2018 | All | 0.953 | (0.947, 0.957) |
| Self-reported BMI | BHIS2018 | All | 0.974 | (0.970, 0.977) |
| Self-reported diabetes | BHIS2018 | Adults | 0.979 | (0.973, 0.983) |
| Self-reported diabetes and following a diet | BHIS2018 | Adults | 0.953 | (0.941, 0.963) |
| Self-reported diabetes and taking a medication | BHIS2018 | Adults | 0.980 | (0.974, 0.984) |
| Self-reported high blood pressure | BHIS2018 | Adults | 0.988 | (0.985, 0.991) |
| Self-reported high blood pressure and following a diet | BHIS2018 | Adults | 0.991 | (0.988, 0.993) |
| Self-reported high blood pressure and taking medication | BHIS2018 | Adults | 0.989 | (0.986, 0.991) |
| Self-reported high cholesterol levels | BHIS2018 | Adults only | 0.990 | (0.987, 0.992) |
| Self-reported high cholesterol levels and following a diet | BHIS2018 | Adults only | 0.973 | (0.966, 0.979) |
| Self-reported high cholesterol levels and taking medication | BHIS2018 | Adults only | 0.991 | (0.988, 0.993) |
| Self-reported myocardial infraction | BHIS2018 | Adults only | 0.980 | (0.974, 0.984) |
| Self-reported coronary heart disease | BHIS2018 | Adults only | 0.987 | (0.984, 0.990) |
| Self-reported other heart serious disease | BHIS2018 | Adults only | 0.991 | (0.989, 0.993) |
| Self-reported stroke | BHIS2018 | Adults only | 0.996 | (0.994, 0.997) |
| Self-reported cancer | BHIS2018 | Adults only | 0.980 | (0.975, 0.984) |
| Measured waist circumference | BELHES2018 | Adults only | 0.475 | (0.404, 0.541) |
| Measured high blood sugar levels | BELHES2018 | Adults only | 0.249 | (0.100, 0.388) |
| Measured systolic blood pressure levels | BELHES2018 | Adults only | 0.653 | (0.606, 0.695) |
| Measured total cholesterol levels | BELHES2018 | Adults only | 0.600 | (0.548, 0.647) |

## Validation of the self-reported data

The risk factor and disease prevalence estimates informed by the BHIS are likely to be subjected to the inaccuracy of self-reporting (i.e., recall bias), as previously acknowledged by the first BELHES (31) as well as the health administrative data from the BCHI (32), and the Belgian national Food Consumption Survey (33). This implies that the true risk factor and disease prevalence could possibly differ from those initially estimated from self-reported data. Hence, the generation of the synthetic population, used as the starting population in the T2D-M, and associated risk factor and disease prevalence at the population level might be limited by the shortcomings of self-reports.

Specifically for this study, the reliability of self-reported information for ascertaining cases of diabetes and hypertension was evaluated by the level of agreement between BHIS and BCHI. In BHIS, self-reported chronic morbidities are defined by the question: “Did you suffer from the indicated disease in the past 12 months?”. The information on this question was compared with drug prescription records available from BCHI considering the Anatomical Therapeutic Chemical (ATC) codes, and Defined Daily Dose (DDD) (**Table 8**), considering a total of at least 90 DDD to assign a certain chronic condition to an individual (34).

A linkage between the BHIS2018 and BCHI2018 databases containing information on 9,753 individuals aged 15 years and older (35), was used to estimate the survey-weighted prevalence of diabetes and hypertension according to the BHIS questionnaire and according to the ATC/DDD-Index. To evaluate the level of agreement between both prevalence estimates, we calculated percentage of agreement between the two prevalence estimates, the Cohen’s kappa coefficient, a measure of interrater reliability, with corresponding 95% bootstrapped confidence intervals, as well as measures of sensitivity, specificity, positive (PPV) and negative predictive value (NPV), taking the BHIS as reference (**Table 9**). Generally, the level agreement is substantial for diabetes (Kappa of 0.80, 95%CI: 0.78, 0.83), but only moderate for hypertension (Kappa of 0.60, 95%CI: 0.58, 0.62), implying that in particular for hypertension using self-reports is likely to underestimate true prevalence, with the caveat that the prevalence estimate from the administrative drug reimbursement data is likely to be an overestimation because antihypertensive medication treatment is also used to treat cardiovascular diseases other than hypertension.

**Table 8** The Anatomical Therapeutic Chemical (ATC) codes assigned to drugs for treating diabetes and hypertension.^1^

| **Condition** | **ATC** | **Medication** |
| --- | --- | --- |
| Diabetes | A10A  A10B | Insulins and analogues  Blood glucose lowering drugs, excluding insulins |
| Hypertension | C02  C03  C07  C08  C09 | Antihypertensive  Diuretics  Beta-blocking agents  Calcium channel blockers  Agents acting on the renin-angiotensin system |

*^1^ Prevalence of chronic conditions was defined by reviewing the medical prescription records using the Anatomical Therapeutic Chemical (ATC) codes, as mentioned here, and the Defined Daily Dose (DDD), following the WHO Collaborating Centre for Drug Statistics Methodology (34). Drugs with more than one active ingredient and with a DDD based on daily prescription not available in the database were assigned with a DDD of 1, while excluded were drugs without information on their DDD, such as pharmacy-prepared drugs. For an individual having an aggregated total of at least 90 DDD for diabetes and/or hypertension drug was considered as presenting the condition.*

**Table 9** Prevalence and measures of agreement between BHIS and BCHI

|  | **Diabetes** | | **Hypertension** | |
| --- | --- | --- | --- | --- |
| Survey-weighted prevalence | BHIS2018 | BCHI2018 | BHIS2018 | BCHI2018 |
|  | 5.9% | 5.8% | 17.6% | 24.6% |
| Agreement (%) | 97.6 | | 86.0 | |
| Kappa (95% CI) | 0.80 (0.78, 0.83) | | 0.60 (0.58, 0.62) | |
| Sensitivity | 80.5 (77.1, 83.5) | | 83.9 (82.1, 85.6) | |
| Specificity | 98.8 (98.6, 99.0) | | 86.5 (85.7, 87.2) | |
| PPV | 82.9 (79.6, 85.9) | | 58.5 (56.5, 60.5) | |
| NPV | 98.6 (98.3, 98.8) | | 95.9 (95.4, 96.4) | |

## Validation of model output

### External validation: Prevalence of risk factors

The prevalence and incidence of hypertension was validated by comparing the use of BP medication using the linkage between BHIS2018 and BCHI data for the years 2018-2020, as previously described. This approach served as validation of the frequency of risk factors for the first years of the simulation among BHIS2018 participants. The ICC values for the comparison of the use of BP medication reported in both databases stratified by age, sex and province, provided overall evidence that the approach of risk factor transition probabilities captures this transition to a similar extent as the baseline year 2018 (**Table 10**). The external validation of other risk factors was not possible due to a lack of comparable external data sources.

**Table 10** External validation of the risk factor BP medication, presented by intra-class correlation coefficient and corresponding 95% confidence intervals of the age-sex-province group estimates, retrieved from the T2D-M and BHIS2018/BCHIS2018-2020.

| **Risk factor** | **Year** | **Population group** | **ICC** | **(95%CI)** |
| --- | --- | --- | --- | --- |
| Prevalence of BP Medication | 2018 | Adults only | 0.444 | (0.325, 0.550) |
|  | 2019 | Adults only | 0.512 | (0.401, 0.608) |
|  | 2020 | Adults only | 0.476 | (0.360, 0.577) |

### External validation: Prevalence and incidence of T2D

The prevalence of T2D was validated using the BHIS2018 linkage with BCHI data from 2018 to 2020. The ICC values for the comparison of the use of T2D prevalence reported in both databases stratified by age, sex and province, provided overall evidence that the T2D-M is capable of modelling the prevalence of T2D-M for the first two years with good reliability (**Table 11**).

**Table 11** External validation of the T2D prevalence, presented by intra-class correlation coefficient and corresponding 95% confidence intervals of the age-sex-province group estimates, retrieved from the T2D-M and BHIS2018/BCHIS2018-2020.

| **Risk factor** | **Year** | **Population group** | **ICC** | **(95%CI)** |
| --- | --- | --- | --- | --- |
| Prevalence of T2D | 2018 | 30-80 years | 0.784 | (0.700, 0.847) |
|  | 2019 | 30-80 years | 0.803 | (0.725, 0.860) |
|  | 2020 | 30-80 years | 0.791 | (0.709, 0.851) |

### External validation: Mortality

The model-estimated mortality rates were compared against the projected mortality rates in Belgium, as forecasted by Federaal Planbureau and STATBEL, for the total population as well as stratified by sex and region (8). Overall, the plots show higher mortality rates for the T2D-M, which might be partly explained by the fact that the model does account for changes in risk factor prevalence that translate into changes in disease incidence and hence mortality.

**Figure 6** Mortality rates, by year, for all ages in total population (A) and stratified by sex (B) and region (C).

Federaal Planbureau and STATBEL projected deaths
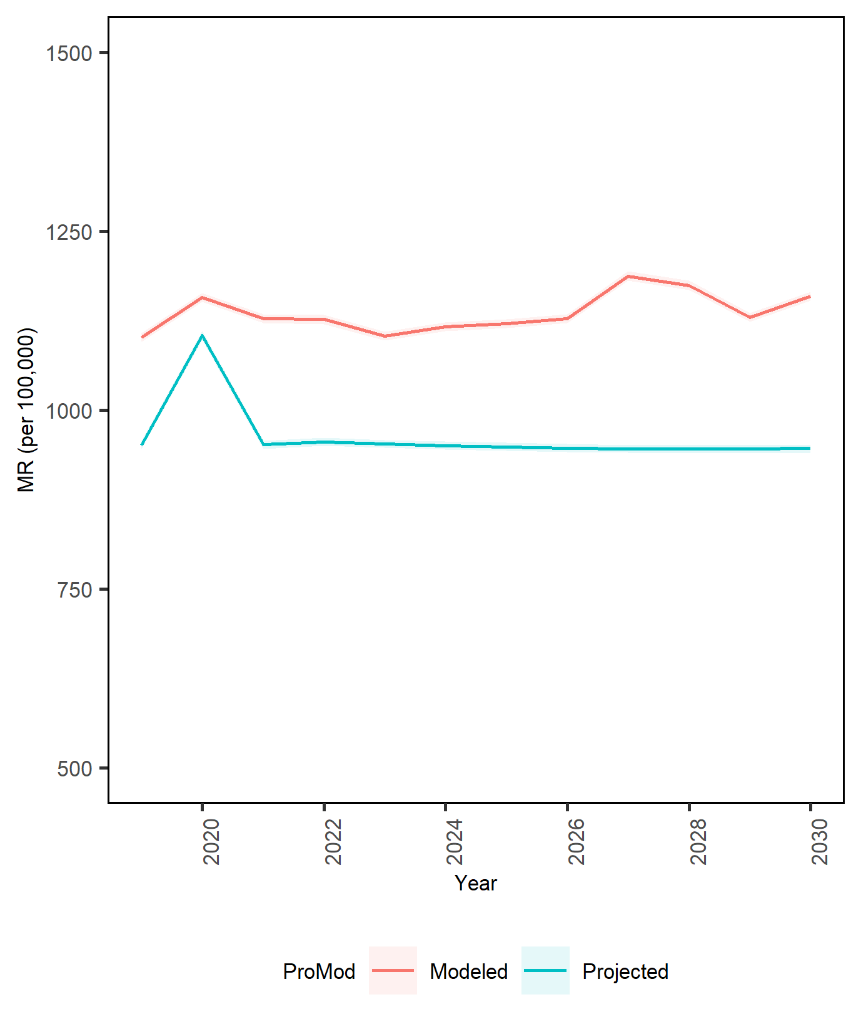
 vs T2D-M modeled deaths
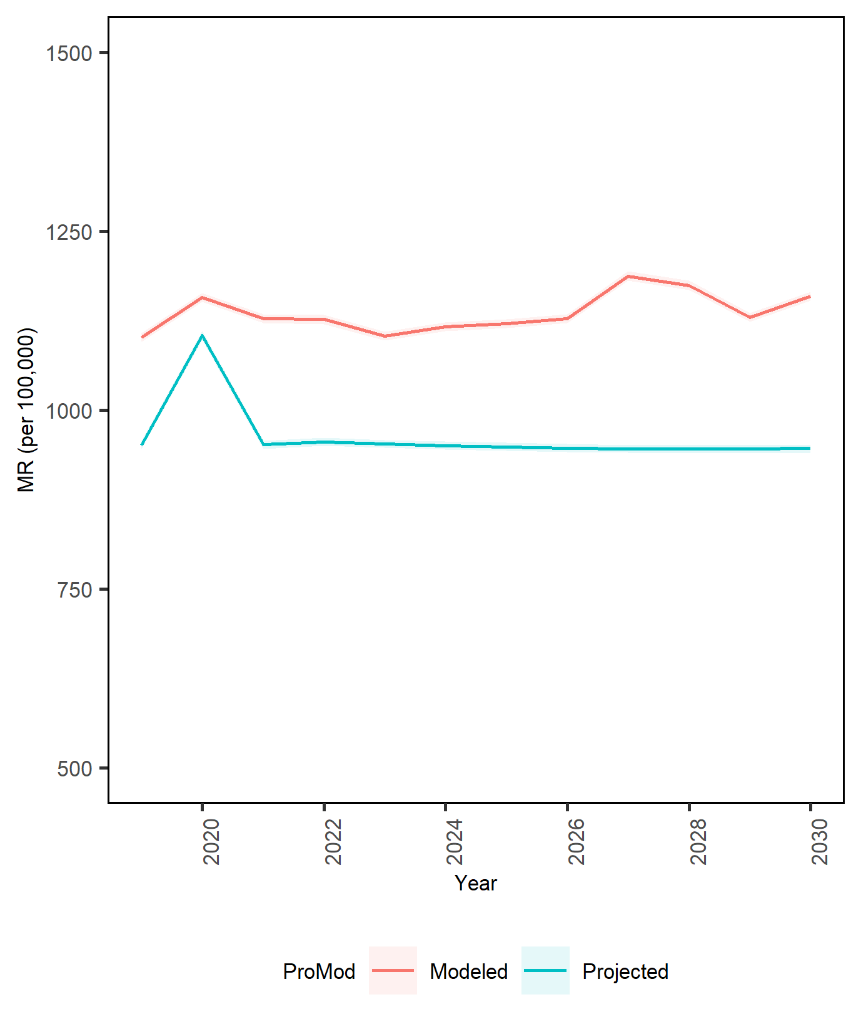


| 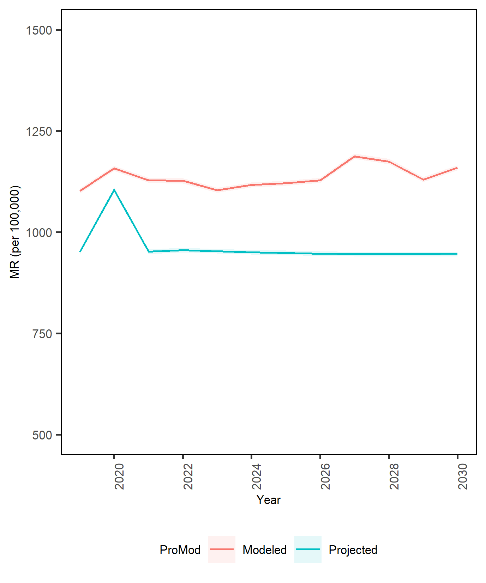 | 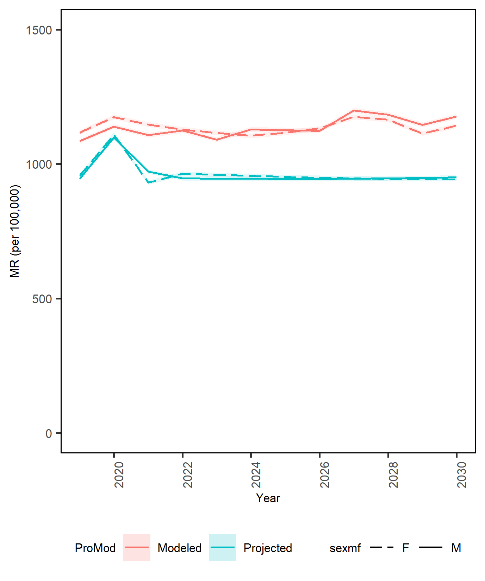 | 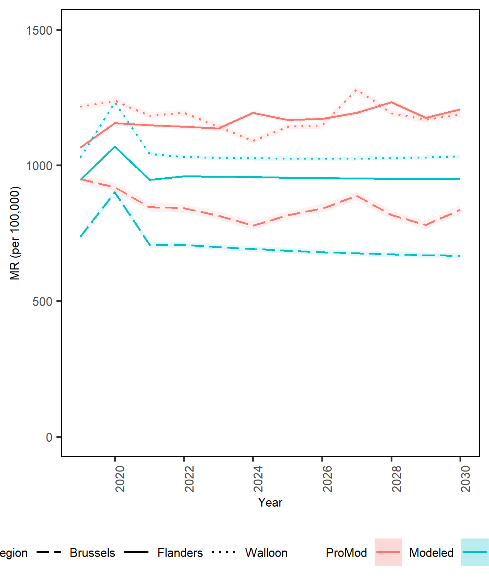 |
| --- | --- | --- |
| A: Total population | B: Stratified by sex:  males 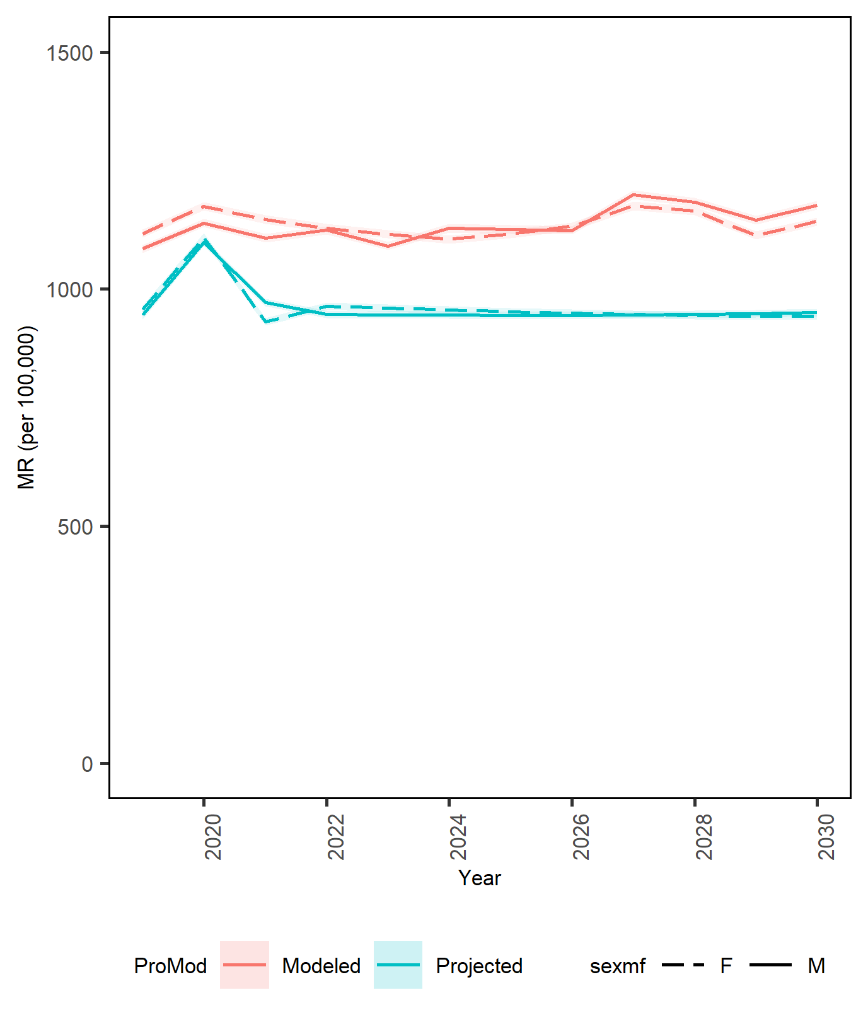and females 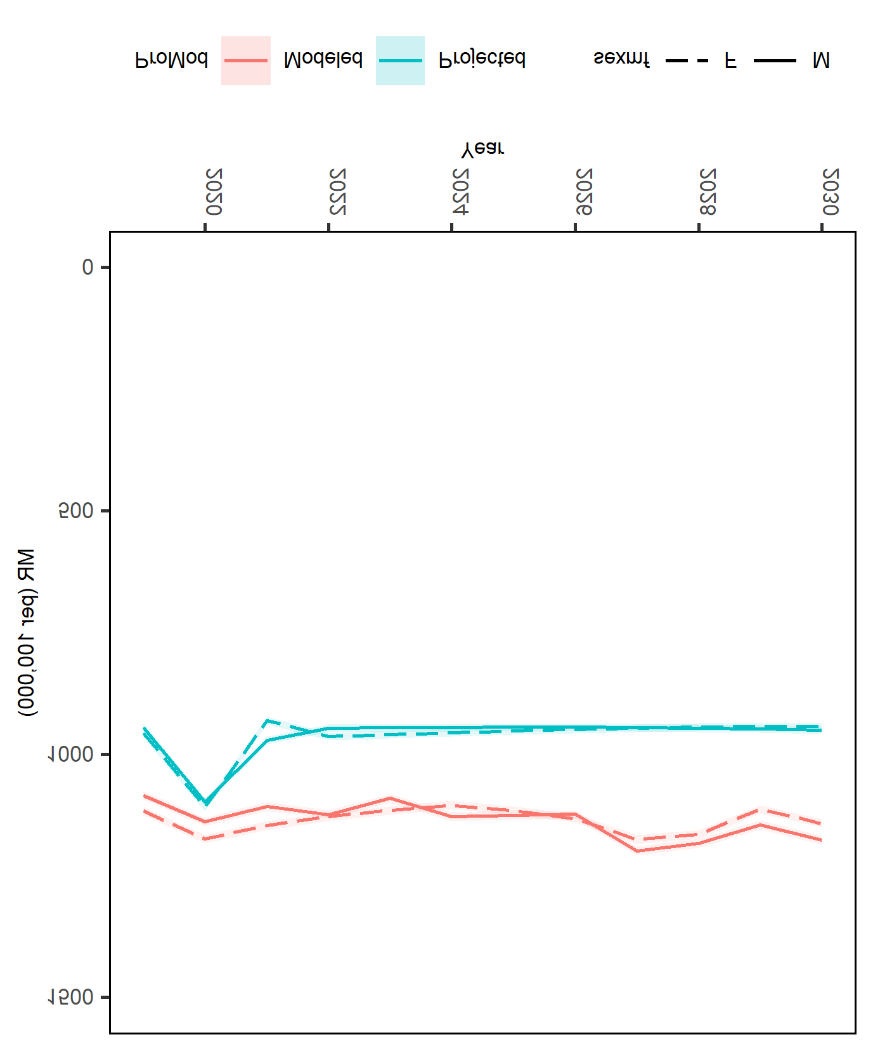 | C: Stratified by region:  Brussels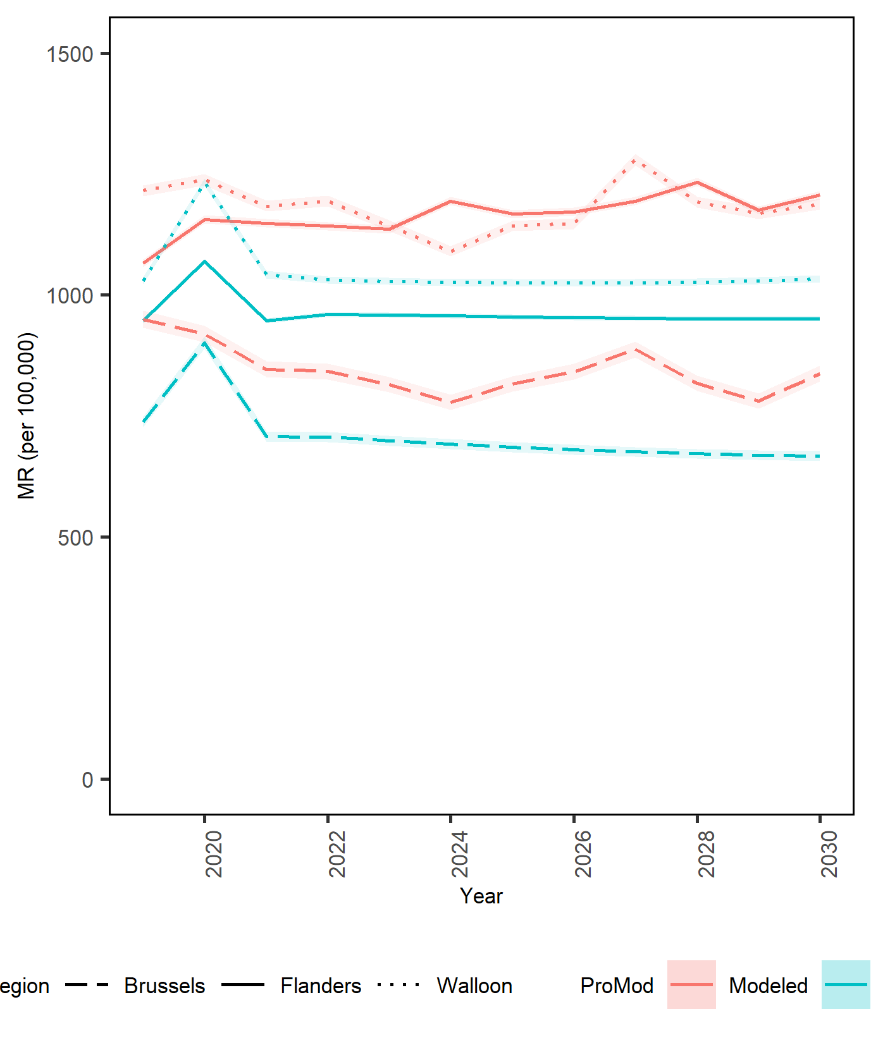 vs Flanders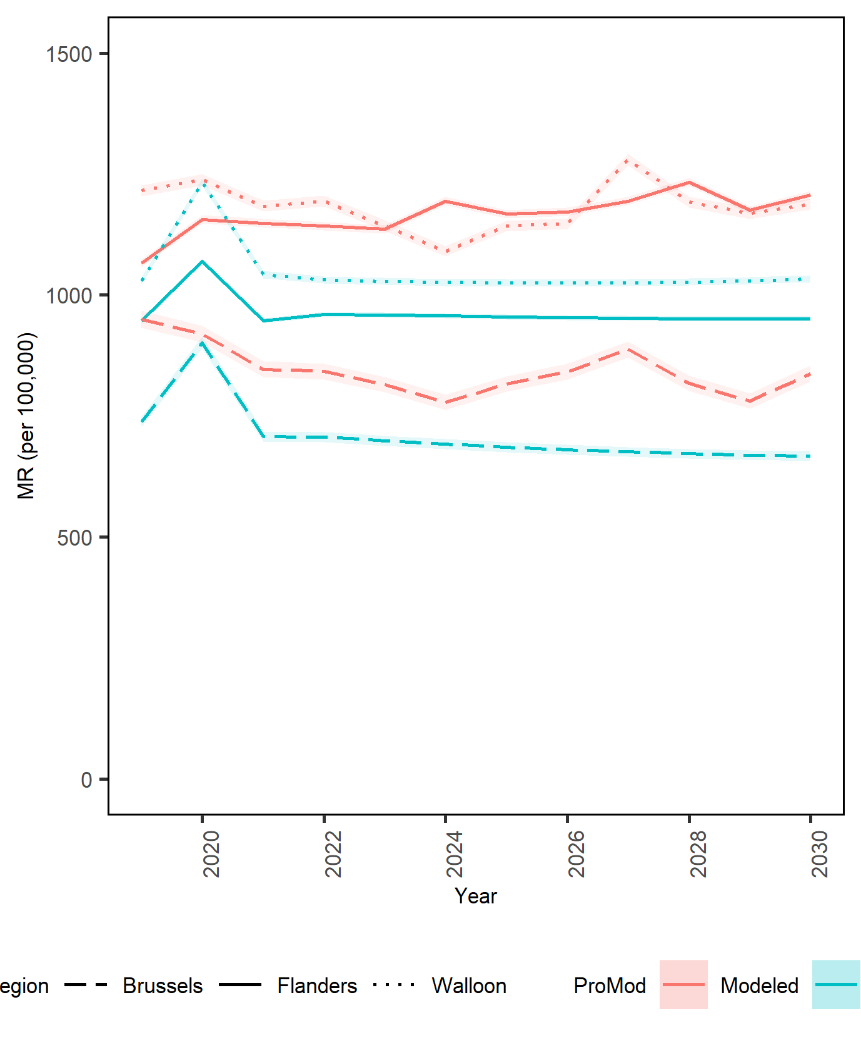 vs Walloon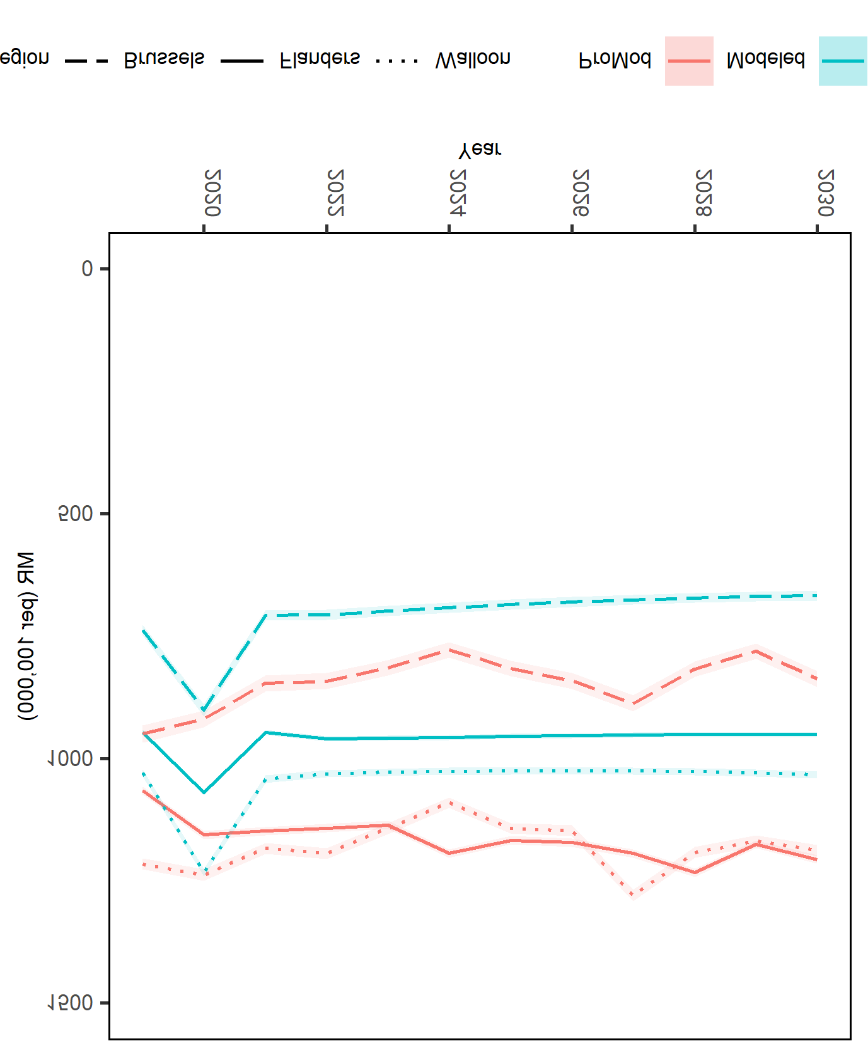 |

# List of assumptions

| Synthetic population |  | - The synthetic population is generated using individual weights (instead of household weights), hence under the assumption of a household of size one. This assumes all individuals in the population are independent (even though household was included as a cluster in the multi-stage stratified population sampling). |
| --- | --- | --- |
| Model structure | Overall | - The model simulates probabilistic transitions through the life-course following a set of subsequential stochastic rules: 1) update of risk factors using strata-specific transition probabilities after increasing age by one year, 2) update T2D status in healthy individuals using probabilities of developing T2D as estimated from the FINDRISC equation, and thereafter 3) update survival status using strata-specific probabilities of death. - The open cohort design introduces new-borns in the synthetic population and accounts for deceased . Migration flows, however, could not be considered because of the lack of information in the survey inputs. |
|  | Age update | - The model simulates the results of aging irrespective of potential influences of socio-demographic changes, since these attributes (gender, province of residence, education and income level) remain the same over the simulation period. |
|  | Risk factor updates | - For children (0-17 years) only BMI updates are simulated until 18 years. At 18 years old, waist circumference is updated based on sex-BMI-specific transition probabilities, while the use of BP medication and history of high blood glucose levels (variables needed for the T2D risk prediction) are assumed not to be present. - For adults, all risk factors are updated via strata-specific transition probabilities. However, due to the small probabilities for BMI and waist circumference, a transition of at least 2.5% of the participants was forced in each year cicle, meaning 97.5% of the individuals, at most, could remain in his/her current risk factor state. - For the use of BP medication and history of high blood glucose the transition to “risk factor not present” was not possible once the risk factor was present. |
|  | T2D status update | - No T2D onsets in children (0-17 years) as the FINDRISC risk equation was not developed for children. Similarly, because FINDRISC was developed primarily for adults aged 35 years and older, age starts to play a role in the T2D-M on ly from 35 years onwards. Hence, model outputs for incident and prevalent T2D are presented for the age group of 35 years onwards. - FINDRISC estimates the 10-year probability for developing T2D, and this was transformed to a one-year probability using the approach described by Fleurence et al. under the assumption of no competing risks. - Incidence of T2D is based on drawing from a binomial distribution using the one-year probability, and once an individual has diabetes (either at baseline or developed over the simulation period), they will have diabetes over the whole simulation period or until they die. |
|  | Survival status update | - Estimated probability of deaths for individuals with diabetes were adjusted using the age-sex-specific relative risk of death from the DECODE study which estimates are not specific for belgium. |
| Model uncertainty | Parameter uncertainty | - In the current implementation, parameter uncertainty considered only the input parameter distribution of the risk prediction equation for developing T2D, i.e., FINDRISC, with parameters equal to the estimated regression coefficients and their confidence limits: a, the minimum value taken from the lower confidence limit, and b, the maximum value taken from the upper confidence limit and minimum values were bounded above 0 when mean above 0 - In contrast, parameter uncertainty for the input parameters of transition probabilities for risk factor states and survival states was considered as relatively small. This is because for the risk factor transition probabilities the mean of 1,000 replicates was used and their 95% CI were narrow, and for mortality probabilities based on smoothing and forecasting with underlying observed mortality data covering more than 30 years. |
| Model validation | External validation | - Prevalence of hypertension using self-reports is likely to underestimate true prevalence, with the caveat that the prevalence estimate from the administrative drug reimbursement data is likely to be an overestimation because antihypertensive medication treatment is also used to treat cardiovascular diseases other than hypertension. - The higher mortality rates for the T2D-M might be partly explained by the fact that our model does account for risk factor prevalence changes that translate in disease incidence and subsequently mortality. |

# References

1. Vlaams Supercomputer Centrum (VSC). Flemish Supercomputer Center (Vlaams Supercomputer Centrum - VSC) User Portal. Brussel, Belgium. 2022. https://www.vscentrum.be/user-portal.

2. van Buuren S, Groothuis-Oudshoorn K. mice: Multivariate Imputation by Chained Equations in R. Journal of Statistical Software. 2011;45(3):1 - 67.

3. Templ M, Meindl B, Kowarik A, Dupriez O. Simulation of Synthetic Complex Data: The R Package simPop. Journal of Statistical Software. 2017;79(10):1 - 38.

4. Buttrey SE. Calling the lp_solve Linear Program Software from R, S-PLUS and Excel. Journal of Statistical Software. 2005;14(4):1 - 13.

5. Eilers PHC, Marx BD. Flexible smoothing with B-splines and penalties. Statistical Science. 1996;11(2):89-121, 33.

6. Hyndman MRJ, Booth H, Tickle L, Maindonald J, Wood S, R Core Team. Package ‘demography’: Forecasting Mortality, Fertility, Migration and Population Data. 2023. https://cran.r-project.org/web/packages/demography/demography.pdf

7. Daniel F, Ooi H, Calaway R, Microsoft, Weston S. Package 'foreach': Provides Foreach Looping Construct. 2022. https://cran.r-project.org/web/packages/foreach/foreach.pdf

8. Belgian Statistical Office (STATBEL). Population theme. 2022. https://statbel.fgov.be/en/themes/population. 2022.

9. Sciensano. BHIS - Belgian Health Interview Surveys. Brussels, Belgium. 2018. https://www.sciensano.be/en/projects/health-interview-survey#protocol-and-questionnaires.

10. Sciensano. BELHES - Belgian Health Examination Surveys. Brussels, Belgium. 2018. https://www.sciensano.be/en/projects/health-examination-survey.

11. Sciensano. SPMA: Standardised Procedures for Mortality Analysis - Belgium. Brussels, Belgium. 2018. https://www.sciensano.be/en/projects/standardized-procedures-mortality-analysis

12. IMA (InterMutualistisch Agentschap). Gezondheidsdata. Brussels, Belgium. 2022. https://ima-aim.be/?lang=nl

13. Lindström J, Tuomilehto J. The diabetes risk score: a practical tool to predict type 2 diabetes risk. Diabetes Care. 2003;26(3):725-31.

14. Roglic G, Unwin N. Mortality attributable to diabetes: estimates for the year 2010. Diabetes Res Clin Pract. 2010;87(1):15-9.

15. Demarest S, Van der Heyden J, Charafeddine R, Drieskens S, Gisle L, Tafforeau J. Methodological basics and evolution of the Belgian health interview survey 1997–2008. Archives of Public Health. 2013;71(1):24.

16. Nguyen D, Hautekiet P, Berete F, et al. The Belgian health examination survey: objectives, design and methods. Archives of Public Health. 2020;78(1):50.

17. Schutte N, Raes L, Devleesschauwer B. Morbidity Statistics. 2019 Pilot Data Collection Belgium, Final Report. Brussels, Belgium: Sciensano. 2020. Report No.: D/2020/14.440/86.

18. De Cocker J, Rietzschel E. Validation of the Finnish diabetes risk score (FINDRISC) for diabetes screening in the Belgian population [MSc thesis]. Gent, Belgium: Ghent University; 2016. https://lib.ugent.be/en/catalog/rug01:002304550?faculty=GE&i=0&lang=dut-und&q=De+Cocker+J&sticky=type-faculty-lang&type=master

19. Alfons A, Kraft S, Templ M, Filzmoser P. Simulation of close-to-reality population data for household surveys with application to EU-SILC. Statistical Methods & Applications. 2011;20(3):383-407.

20. Leys C, Ley C, Klein O, Bernard P, Licata L. Detecting outliers: Do not use standard deviation around the mean, use absolute deviation around the median. Journal of Experimental Social Psychology. 2013;49(4):764-6.

21. World Health Organisation (WHO) Mutlicentre Growth Reference Study. WHO Child Growth Standards: length/height-for-age, weight-for-age, weight-for-length, weight-for-height and body mass index-for-age: methods and development. Geneva. 2006.

22. de Onis M, Onyango AW, Borghi E, Siyam A, Nishida C, Siekmann J. Development of a WHO growth reference for school-aged children and adolescents. Bull World Health Organ. 2007;85(9):660-7.

23. Statbel (the Belgian Statistical Office). Population Projections: population trends per province 1992-2070 (in Dutch: Bevolkingsvooruitzichting: loop van de bevolking per provincie 1992-2070). Brussels , Belgium- 2020.

24. Kassteele J, Hoogenveen RT, Engelfriet PM, Baal PH, Boshuizen HC. Estimating net transition probabilities from cross-sectional data with application to risk factors in chronic disease modeling. Stat Med. 2012;31(6):533-43.

25. Fleurence RL, Hollenbeak CS. Rates and probabilities in economic modelling: transformation, translation and appropriate application. Pharmacoeconomics. 2007;25(1):3-6.

26. Hyndman RJ, Ullah MS. Robust forecasting of mortality and fertility rates: A functional data approach. Comput. Stat. Data Anal. 2007;51(10):4942–56.

27. De Pauw R, Gorasso V, Devleesschauwer B. Belgian national burden of disease study. Guidelines for the calculation of DALYs in Belgium: Sciensano. 2022 Contract No.: D/2022/14.440/09.

28. Global Burden of Disease Collaborative Network. Global Burden of Disease Study 2019 (GBD 2019) Disability Weights. Seattle, WA, USA: Institute for Health Metrics and Evaluation (IHME). 2020.

29. Lavens A, De Block C, Mathieu C, et al. Initiative pour la Promotion de la Qualité et l’Epidémiologie du Diabète sucré. Bruxelles, Belgique: Sciensano. 2022 Contract No.: D/2022/14.440/26.

30. Briggs AH, Weinstein MC, Fenwick EA, Karnon J, Sculpher MJ, Paltiel AD. Model parameter estimation and uncertainty analysis: a report of the ISPOR-SMDM Modeling Good Research Practices Task Force Working Group-6. Med Decis Making. 2012;32(5):722-32.

31. J. Van der Heyden DN, F. Renard, A. Scohy, S. Demarest, S. Drieskens, L. Gisle. Belgische gezondheidsonderzoek 2018. Brussel, België: Sciensano. 2019 Contract No.: Rapportnummber: 2019/14.440/89.

32. Berete F, Demarest S, Charafeddine R, Bruyère O, Van der Heyden J. Comparing health insurance data and health interview survey data for ascertaining chronic disease prevalence in Belgium. Archives of Public Health. 2020;78(1):120.

33. Drieskens S, Demarest S, Bel S, De Ridder K, Tafforeau J. Correction of self-reported BMI based on objective measurements: a Belgian experience. Archives of Public Health. 2018;76(1):10.

34. WHO Collaborating Centre for Drug Statistics Methodology (WHOCC). International language for drug utilization research. WHO Collaborating Centre for Drug Statistics Methodology, Oslo, Norway. 2022.

35. eGenzonheid. Informatieveiligheidscomité Kamer sociale zekerheid en gezondheid. Brussels, Belgium2016. https://www.ehealth.fgov.be/ehealthplatform/file/view/AXW7dlaDl9vUUfvGGe52?filename=20-204-n382-HISLINK%202018.pdf
